# Elucidating the material basis and potential mechanisms of Ershiwuwei Lvxue Pill acting on rheumatoid arthritis by UPLC-Q-TOF/MS and network pharmacology

**DOI:** 10.1371/journal.pone.0262469

**Published:** 2022-02-07

**Authors:** Chuan Liu, Fangfang Fan, Lu Zhong, Jinsong Su, Yi Zhang, Ya Tu

**Affiliations:** 1 School of Food and Bioengineering, Xihua University, Chengdu, China; 2 Ethnic Medicine Academic Heritage Innovation Research Center, Chengdu University of Traditional Chinese Medicine, Chengdu, China; 3 Development Research Center of Traditional Chinese Medicine, China Academy of Traditional Chinese Medicine, Beijing, China; Tianjin University of Traditional Chinese Medicine, CHINA

## Abstract

Ershiwuwei Lvxue Pill (ELP, མགྲིན་མཚལ་ཉེར་ལྔ།), a traditional Tibetan medicine preparation, has been used hundreds of years for the clinical treatment of rheumatoid arthritis (RA) in the highland region of Tibet, China. Nevertheless, its chemical composition and therapeutic mechanism are unclear. This study aimed to uncover the potentially effective components of ELP and the pharmacological mechanisms against RA by combing UPLC-Q-TOF/MS and network pharmacology. In this study, 96 compounds of ELP were identified or tentatively characterized based on UPLC-Q-TOF/MS analysis. Then, a total of 22 potential bioactive compounds were screened by TCMSP with oral bioavailability and drug-likeness. Preliminarily, 10 crucial targets may be associated with RA through protein-protein interaction network analysis. The functional enrichment analysis indicated that ELP exerted anti-RA effects probably by synergistically regulating many biological pathways, such as PI3K-Akt, Cytokine-cytokine receptor interaction, JAK-STAT, MAPK, TNF, and Toll-like receptor signaling pathway. In addition, good molecular docking scores were highlighted between five promising bioactive compounds (ellagic acid, quercetin, kaempferol, galangin, coptisine) and five core targets (PTGS2, STAT3, VEGFA, MAPK3, TNF). Overall, ELP can exert its anti-RA activity via multicomponent, multitarget, and multichannel mechanisms of action. However, further studies are needed to validate the biological processes and effect pathways of ELP.

## Introduction

Ershiwuwei Lvxue Pill (ELP, མགྲིན་མཚལ་ཉེར་ལྔ།), a traditional Tibetan patented prescription medicine, was officially recorded in the Drug Standard of the Ministry of Public Health of the Peoples Republic of China for the treatment of rheumatoid arthritis (RA) [1]. According to Tibetan medicine theory, it was one of the traditionally used drugs to cure gout and arthralgia, and demonstrated to effectively cure RA in clinical trials [2]. ELP is composed of 25 herbs, mineral salts or animal drugs, namely *Equus asinus* Linnaeus, *Rhamnella gilgitica* Mansf. et Melch, *Dalbergia odorifera* T.C.Chen, *Santalum album* L, *Terminalia bellirica* (Gaertn.) Roxb, *Terminalia chebula* Retz, Calcareous tuff, *Phyllanthus emblica* L, *Myristica fragrans* Houtt, *Eugenia caryophyllata* Thunb, *Amomum tsao-ko* Crevost & Lemarié, *Amomum kravanh* Pierre ex Gagnep, *Cassia obtusifolia* L, *Boswellia carteri* Birdw, *Gossampinus malabarica* (DC.) Merr, *Abelmoschus manihot* (L.) Medik, *Pterocephalus hookeri* (C.B.Clarke) Hoeck, *Gentiana manshurica* Kitag, *Saxifraga pasumensis* Marg.et Shwa, *Adhatoda vasica* Nees, *Tinospora sinensis* (Lour.) Merr, *Fraxinus rhynchophylla* Hance, *Moschus berezovskii* Flerov, *Crocus sativus* L, *Bos taurus domesticus* Gmelin (S1 Table). The complex chemical compositions of ELP mainly consists of phenolic acids, flavonoids, and alkaloids, such as ellagic acid, gallic acid, quercetin, kaempferol, coptisine, and jatrorrhizine, which have been reported to possess analgesic, anti-inflammatory, and antimicrobial effects [3–6]. In addition, previous studies have suggested that the anti-RA effect of ELP may be related to the reduction of serum levels of Interleukin (IL)-17α in collagen Ⅱ-induced arthritis (CIA) rats [7]. Nonetheless, the shortcomings limit the further study of ELP: firstly, the clear relationship between ingredients and herbs has not been scientifically established, largely due to its complex compound composition; secondly, in terms of the research on the drug action mechanism, it is difficult to accurately reflect the multi-component, multi-target and multi-pathway characteristics of Tibetan medicine formula only by a single active ingredient or indicator component [8,9]. All those obstacles have hampered the efficacy evaluation and secondary development of Tibetan medicine ELP.

At present, a system pharmacological approach integrating Ultra-performance liquid chromatography coupled to quadrupole time-of-flight mass spectrometry (UPLC-Q-TOF/MS) and network pharmacology with molecular docking simulation has been widely used in studies on the efficacious material basis and mechanism of action of Traditional Chinese medicine or Tibetan medicine [10,11]. Given the technical merit of high sensitivity, selectivity, and speed of information collection, UPLC-Q-TOF/MS can effectively solve the problems such as complex components and difficult quantification in the modern analysis of natural medicine and has outstanding advantages in the rapid discovery and characterization of target components [12,13]. Network pharmacology transforms a “one-target, one-drug” model into a “multicomponent, multitarget” model, which effectively illustrates the complex interaction between disease and drugs from the network perspective and has been widely used in mechanism research in Tibetan medicine [14,15]. In addition, molecular docking simulation is a method of matching a ligand (drug) to a target molecule (receptor) by producing various components in different directions, as is an excellent tool in drug discovery perspective and drug molecular design [16,17]. Interestingly, the successful combination of molecular docking simulation and network pharmacology in Traditional Chinese medicine/Tibetan medicine pharmacology provides a powerful model for the treatment of RA with ELP [18,19].

In this study, 96 compounds were identified or tentatively characterized based on UPLC-Q-TOF/MS analysis. Then, combining network pharmacology and molecular docking simulation, the potential biologically active compounds, targets, and underlying mechanisms of ELP for the treatment of RA were investigated.

## Materials and methods

### Materials and chemicals

ELP was obtained from Tibetan mandew Tibetan Medicine Co. Ltd (Tibet, China), lot number: 19103A. It is a brown water pellet with a diameter of about 0.8 cm, which is fragrant and sour in taste. The proportion of each component in ELP was shown in the S1 Table. HPLC grade acetonitrile and formic acid were supplied by Fisher Scientific (Fisher, Fair Lawn, NJ, USA). Chemical reference standards of gallic acid (110831–201906), chlorogenic acid (110753–201817), esculetin (110741–201708), ellagic acid (11959–201602), rutin (100080–201811), quercetin (100081–200907), and oleanolic acid (110709–201808) were purchased from National Institute for the Control of Pharmaceutical and Biological Products (Beijing, China). Corilagin (K-004-190509), orientin (H-044-181216), and isovitexin (Y-116-180803) were provided from Chengdu Ruifensi Biotechnology Co., Ltd. (Chengdu, China). Loganic acid (CHB190109), chebulinic acid (CHB190724), crocin I (CHB190308), ursodeoxycholic acid (CHB180626), (+)-Bicuculline (CHB190112), and β-boswellic acid (CHB190625) were purchased from Chengdu Chroma-Biotechnology Co., Ltd. (Chengdu, China). The purity of the standards was above 98%.

### Sample preparation

Accurately-weighed ELP sample (0.5 g) was placed in a conical flask for ultrasound-assisted extraction with 70% methanol (30 mL) at 25°C for 30 min. The extracted solution was adjusted to the original weight by adding 70% methanol, and then the extracted solution was centrifuged at 14 000 rpm for 5 min. The supernatant was filtered through a 0.22 μm microporous membrane filter prior to injection into the UPLC-Q-TOF/MS system. Standard stock solutions of the reference standards were prepared by dissolving appropriate amounts of the pure substances in methanol.

### UPLC-Q-TOF/MS analysis

A qualitative analysis of ELP was performed by UPLC (Waters, Milford, MA, USA). The chromatographic separation was performed on an Acquity UPLC BEH C18 column (2.1 mm × 100 mm, 1.7 μm, Waters), and the temperature was settled at 35°C. The mobile phase was composed of acetonitrile (A) and 0.1% aqueous formic acid solution (B), and the flow rate was 0.4 mL/min. The linear gradient program was performed as follows: 0–1 min, 5% A; 1–3.5 min, 5–20% A; 3.5–4.5 min, 20–25% A; 4.5–6 min, 25–30% A; 6–9 min, 30–50% A; 9–11 min, 50–75% A; 11–13 min, 75–90% A;13–15 min, 90–95% A;15–16 min, 95% A; 16–16.5 min, 95–5% A;16.5–19 min, 5% A.

Xevo G2-XS Q-TOF (Waters, Manchester, UK) equipped with an electrospray ionization ion (ESI) source was applied for mass spectrometry data’s acquisition in negative and positive ionization mode ranging from m/z 100 to m/z 1200. The parameters of the source were set as follows: the ion source temperature was set at 120°C, cone voltage 25 V, capillary voltage 2.5 kV, collision energy 10 V, desolvation temperature 350°C, and desolvation gas flow rate, 1000 L/h.

### Acquisition of active compounds and their targets

All compounds obtained by UPLC-Q-TOF/MS analysis were retrieved based on the absorption, distribution, metabolism, and excretion (ADME) parameters in the Traditional Chinese Medicine Systems Pharmacology Database and Analysis Platform (TCMSP, http://lsp.nwu.edu.cn/tcmsp.php), the following screening criteria were used: oral bioavailability (OB) ≥30%, drug-likeness (DL) ≥0.18 [20]. Then, the selected bioactive compounds were converted to a standard Canonical Simplified molecular input line entry system (SMILES) format in PubChem database (https://pubchem.ncbi.nlm.nih.gov/), at the same time, SMILES format file was imported to Swiss Target Prediction database with properties for "Homo sapiens", and eventually ELP potential targets were built in the bioactive compounds information database [21].

### Acquisition of targets for RA

Different genes related to RA were obtained from the Comparative Toxico genomics Database (CTD, http://ctdbase.org/) [22], GeneCards HUMAN GENE DATABASE (https://www.genecards.org/) [23], and Online Mendelian Inheritance in Man (OMIM, http://www.omim.org/) [24]. The keyword “rheumatoid arthritis” was used to search for RA-related targets in CTD, GeneCards, and OMIM databases.

### Protein-Protein Interaction Data

Protein-Protein Interaction (PPI) Data were extracted using STRING (https://string-db.org/) with a reasonable confidence range for PPI data scores (low: <0.4, medium: 0.4 to 0.7, and high: >0.7) [25]. Common targets of bioactive compounds associated with RA were inputted into the STRING database, with the species to “Homo sapiens,” and a confidence score higher than 0.7. The visualization of the PPI networks was conducted in Cytoscape 3.8.0 software (http://www.cytoscape.org/) [26].

### Functional enrichment analysis

Based on potential common targets for this study, GeneOntology (GO) and Kyoto Encyclopedia of Genes and Genomes (KEGG) analyses were conducted to predict the action mechanism of ELP in treating RA by using the Database for Annotation, Visualization, and Integrated Discovery (DAVID v6.8, https://david.ncifcrf.gov/) [27].

### Compound-Target-Pathway (C-T-P) network

Based on the results in the DAVID database, the C-T-P network was created in Cytoscape 3.8.0 software. In a complex associative network, nodes and edges represent compounds/entities and their direct interactions, respectively. A high degree value equates to a prominent node status.

### Molecular docking simulation

The molecular docking simulation was performed on the selected targets and corresponding compounds by using Maestro version 11.5 from the Schrodinger software suite. The lowest/minimum energy conformation was used for molecular docking via the default parameters. The docking score is the negative logarithm of the experimental dissociation/inhibition constant (pKd/pKi) and usually ranges from 0 to 10 (weak to strong combination force) [28]. The human protein structures with the highest resolution related to RA were selected from the UniProt database (https://www.uniprot.org/). Meanwhile, the X-ray crystal structures of these proteins were obtained from the RCSB PDB database (https://www.rcsb.org/).

## Results

### Identification of chemical compounds in ELP by UPLC-Q-TOF/MS

**Fig 1:**
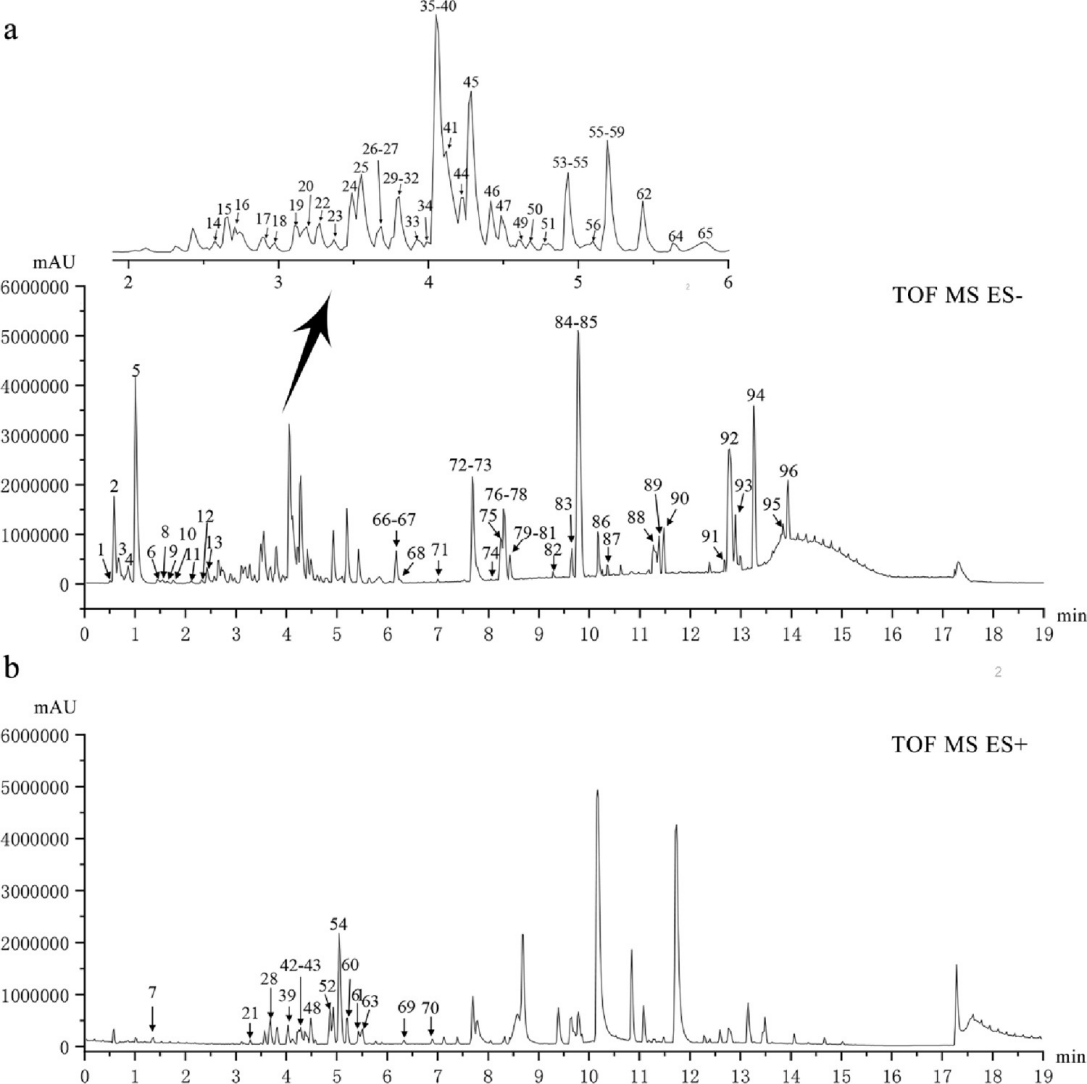
UPLC-Q-TOF-MS total ion chromatogram of ELP. (A) negative mode; (B) positive mode.

A total of 96 compounds were identified based on UPLC-Q-TOF/MS in the positive and negative ion mode, and the total ion chromatogram was analyzed based on the chemical standard, fragmentation patterns, previous literature data, and the UNIFI software (v1.8, Waters Corp., Milford, MA, USA) (Figs 1 and S1). Meanwhile, the following public databases were consulted: ChemSpider (http://www.chemspider.com), SciFinder Scholar (https://scifinder.cas.org).

According to various structures, tannins include phenolic acids, simple gallic acid esters, and ellagitannins. The basic structural units of tannin compounds are gallic acid and glucose, which contain or lose -C_7_H_5_O_5_ (m/z 169), -C_7_H_4_O_4_ (m/z 152), -C_6_H_5_O_3_ (m/z 125) ion fragments in the MS/MS spectrum. Ellagitannins, a hydrolyzable tannin class of polyphenols, hydrolyzed with acids or bases to produce hexahydroxy diphenolic acid (HHDP), which were further spontaneously esterified into ellagic acid [29]. ESI-MS analysis of flavonoids exhibited some diagnostic features such as loss of methyl, methoxyl, H_2_O (18 Da), CO (28 Da), CO_2_ (44 Da), C_2_H_2_O (42 Da) [30]. Meanwhile, for the identification of flavonoid aglycones, the retro-Diels-Alder (RDA) reaction provides structurally relevant information on the number and types of substituents in the A- and B-rings of the two C-C bonds in Cleavage of the C-ring [31]. In addition, flavonoids C-glycosides in ESI-MS/MS spectra, ions of [M-H-C_2_H_4_O_2_ (60 Da)]^-^, [M-H-C_3_H_6_O_3_ (90 Da)]^-^, [M-H-C_4_H_8_O_4_ (120 Da)]^-^ are considered as characteristic of C-glycoside flavonoids under low collision energy. Alkaloids are a kind of basic organic compounds containing nitrogen, which have a good response in ESI (+) mode and mainly appear in the positive mode as [M+H]^+^ [32].

Finally, in the positive ion mode, 14 chromatographic peaks, mainly alkaloids, were characterized; and in the negative ion mode, 82 chromatographic peaks were identified, mainly 22 tannins, 17 flavonoids, 3 penylpropanoids, 23 terpenoids, 3 quinonoids, 3 steroids, and 11 other compounds (Table 1). The fragmentation pathways of representative compounds from ELP (S2 Fig).

**Table 1:** Characterization of the chemical constituents in ELP by UPLC-Q-TOF-MS.

NO.	Rt (min)	Compound	Molecular formula	Molecular weight (Da)	Detected ion (m/z) [M-H]^-^/[M+H]^+^	Error (ppm)	MS^2^ fragment ion (m/z)
1	0.53	Arginine	C_6_H_14_N_4_O_2_	174.1117	173.1041	-1.5	-
2	0.61	Quinic acid	C_7_H_12_O_6_	192.0634	191.0561	-0.3	173.0456[M-H-H_2_O]^-^, 127.907[M-H-2H_2_O-CO]^-^
3	0.78	Chebulic acid	C_14_H_12_O_11_	356.038	355.031	0.8	337.0201[M-H-H_2_O]^-^, 293.0590[M-H-H_2_O-CO_2_]^-^, 275.0197[M-H-2H_2_O-CO_2_]^-^
4	0.80	Monogalloyl glucose	C_13_H_16_O_10_	332.0743	331.0674	-0.9	313.0020[M-H-H_2_O]^-^, 271.0459[M-H-C_2_H_4_O_2_]^-^, 211.0951[M-H-C_4_H_8_O_4_]^-^, 169.0142[M-H-glc]^-^, 124.9060[M-H-glc-CO_2_]^-^
5	1.00	Gallic acid*	C_7_H_6_O_5_	170.0215	169.0142	0.9	125.0243[M-H-CO_2_]^-^, 107.0132[M-H-CO_2_-H_2_O]^-^
6	1.28	Punicalin	C_34_H_22_O_22_	782.0603	781.0515	2.9	600.9894[M-H-glc]^-^, 300.8809[DPPH]
7	1.33	N-Methyl-2,3-dioxole-Tetrahydroisoquinoline	C_11_H_13_NO_2_	191.0946	192.0992	-3.3	177.0765[M+H-CH_3_]^+^, 149.0807[M+H-CH_3_-CO]^+^
8	1.55	5-Galloylshikimic acid	C_14_H_14_O_9_	326.0638	325.0566	1.8	169.0140[gallic acid-H]^-^, 153.0200, 125.0244[gallic acid-H-CO_2_]^-^
9	1.63	Phenylalanine	C_9_H_11_NO_2_	165.079	164.0721	0.9	-
10	2.11	1,2-di-O-galloyl-D-glucose	C_20_H_20_O_14_	484.0853	483.0776	-1.5	331.0671[M-H-galloyl]^-^, 313.0562[M-H-galloyl-H_2_O]^-^, 271.0448, 211.0246, 169.0139[gallic acid-H]^-^
11	2.13	Glucosyringic Acid	C_15_H_20_O_10_	360.1057	359.0981	0.8	197.0452[M-H-glc]^-^, 153.0535[M-H-glc-CO_2_]^-^
12	2.23	Neochlorogenic acid	C_16_H_18_O_9_	354.0951	353.0879	1.5	191.0553[M-H-caffeoyl]^-^, 179.0349[caffeic acid-H]^-^, 173.0480[M-H-caffeoyl-H_2_O]^-^, 135.0492[caffeic acid-H-CO_2_]^-^
13	2.52	Esculin	C_15_H_16_O_9_	340.0794	339.0716	0	177.0189[M-H-glc]^-^, 149.0711[M-H-glc-CO]^-^, 133.0295[M-H-glc-CO_2_]^-^, 121.0625[M-H-glc-CO-CO]^-^,
14	2.58	Digalloylglucose isomer I/2,3-di-O-galloyl-D-glucose	C_20_H_20_O_14_	484.0853	483.0776	-1.5	331.0673, 313.0565, 271.0456, 211.0247, 169.0139
15	2.65	Digalloylglucose isomer II/3,6-di-O-galloyl-D-glucose	C_20_H_20_O_14_	484.0853	483.0779	-1.5	331.0671, 313.0563, 271.0458, 211.0245, 169.0139
16	2.72	Loganic acid*	C_16_H_24_O_10_	376.1369	375.1297	-0.8	213.0766[M-H-glc]^-^, 169.0864[M-H-glc-CO_2_]^-^, 151.0761[M-H-glc-CO_2_-H_2_O]^-^,
17	2.89	Chlorogenic acid*	C_16_H_18_O_9_	354.0951	353.0877	1.5	191,0554[M-H-caffeoyl]^-^, 179.0347[Caffeic acid-H]^-^
18	3.11	Lutonarin	C_27_H_30_O_16_	610.1534	609.1458	1.8	447.0933[M-H-glc]^-^, 357.0616[M-H-glc-90]^-^, 327.0512[M-H-glc-120]^-^,
19	3.12	Esculetin*	C_9_H_6_O_4_	178.0266	177.0194	-0.7	149.0240[M-H-CO]^-^, 133.0288[M-H-CO_2_]^-^, 121.0732[M-H-CO-CO]^-^
20	3.16	Isovitexin-7-O- glucoside	C_27_H_30_O_15_	594.1584	593.1512	1.3	503.1197[M-H-90]^-^, 473.1085[M-H-120]^-^, 341.0661[M-H-glc-90]^-^, 311.0551[M-H-glc-120]^-^,
21	3.17	N-trans Feruloyltyramine	C_18_H_19_NO_4_	313.1314	314.1368	-0.6	299.1100[M+H-CH_3_]^+^, 177.0520, 121.0646[C_8_H_9_O]^+^
22	3.19	1,2,6-Trigalloylglucose	C_27_H_24_O_18_	636.0963	635.0891	-1.9	483.0780[M-H-galloyl]^-^, 465.1779[M-H-galloyl-H_2_O]^-^, 331.1322[M-H-2galloyl]^-^, 313.0762[M-H-2galloyl-H_2_O]^-^, 178.9124[M-H-3galloyl]^-^,
23	3.29	Genipin-1-O-Gentiobioside	C_23_H_34_O_15_	550.1898	549.1807	-1.2	517.1517[M-H-CH_3_OH]^-^, 323.0980 [M-H-genipin]^-^, 225.0791[M-H-2glc]^-^, 207.0599[M-H-2glc-H_2_O]^-^, 123.0478 [M-H-2glc-H_2_O-C_4_H_4_O_2_]^-^
24	3.37	Corilagin*	C_27_H_22_O_18_	634.086	633.0733	2.2	481.0671[M-H-galloyl]^-^, 463.0520[M-H-galloyl-H2O]^-^, 300.9992[M-H-galloyl-H_2_O-Hex]^-^
25	3.40	Cryptochlorogenic acid	C_16_H_18_O_9_	354.0951	353.0887	1.5	191.0553[M-H-caffeoyl]^-^, 179.0349[caffeic acid-H]^-^, 173.0480[M-H-caffeoyl-H_2_O]^-^, 135.0492[caffeic acid-H-CO_2_]^-^
26	3.49	1,2,6-Trigalloylglucose	C_27_H_24_O_18_	636.0963	635.0891	-1.9	483.0780[M-H-galloyl]^-^, 465.1779[M-H-galloyl-H_2_O]^-^, 331.1322[M-H-2galloyl]^-^, 313.0762[M-H-2galloyl-H_2_O]^-^, 178.9124[M-H-3galloyl]^-^,
27	3.55	1,2,6-Trigalloylglucose	C_27_H_24_O_18_	636.0963	635.0891	-1.9	483.0780[M-H-galloyl]^-^, 465.1779[M-H-galloyl-H_2_O]^-^, 331.1322[M-H-2galloyl]^-^, 313.0762[M-H-2galloyl-H_2_O]^-^, 178.9124[M-H-3galloyl]^-^,
28	3.68	Isocorydine	C_20_H_23_NO_4_	341.1627	342.1706	0.1	311.1422[M+H-CH_3_NH_2_]^+^, 297.1134[M+H-(CH_3_)_2_NH_2_]^+^, 279.1079[M+H-CH_3_NH_2_-CH_3_OH]^+^
29	3.69	Fraxetin	C_10_H_8_O_5_	208.0372	207.0297	-0.8	192.0062[M-H-CH_3_]^-^, 164.0111[M-H-CH_3_-CO]^-^,
30	3.81	Orientin*	C_21_H_20_O_11_	448.1006	447.0933	0.5	357.0618[M-H-90]^-^, 327.0511[M-H-120]^-^,
31	3.83	Cajanin	C_16_H_12_O_6_	300.0634	299.0568	1.2	284.0324[M-H-CH_3_]^-^ (100%), 256.0[M-H-CH_3_-CO]^-^,
32	3.92	Chebulagic acid	C_41_H_30_O_27_	954.0975	953.0884	-0.2	454.2341[M-H-COOH]^2-^, 476.3053 [M-2H]^2-^, 300.9994[HHDP-H]^-^,
33	3.99	1,2,3,6-Tetragalloylglucose	C_34_H_28_O_22_	788.1072	787.1003	0.9	635.0890[M-H-galloyl]^-^, 483.0776[M-H-2galloyl]^-^, 331.0931[M-H-3galloyl]^-^,
34	4.01	Maesopsin	C_15_H_12_O_6_	288.0637	287.0559	1.1	269.0461[M-H-H_2_O]^-^, 259.0622[M-H-CO]^-^, 215.0702[M-H-CO-CO_2_]^-^
35	4.03	Kaempferol-3-O-gentiobioside	C_27_H_30_O_16_	610.1534	609.1461	1.8	285.0783[M-H-2glc]^-^, 267.0585[M-H-2glc-H_2_O]-;
36	4.05	Syringic acid	C_9_H_10_O_5_	198.0528	197.0456	0.8	182.0568[M-H-CH_3_]^-^, 179.0347[M-H-H_2_O]^-^, 135.0033[M-H-CO_2_]^-^
37	4.09	1,3,4,6-Tetragalloylglucose	C_34_H_28_O_22_	788.1072	787.1003	0.9	635.0890[M-H-galloyl]^-^, 483.0776[M-H-2galloyl]^-^, 331.0931[M-H-3galloyl]-,
38	4.39	Ellagic acid*	C_14_H_6_O_8_	302.0063	300.9973	-1.1	283.9963[M-H- H_2_O]^-^, 273.0189[M-H-CO]^-^, 257.0082[M-H-CO2]^-^, 229.0141[M-H-CO_2_-CO]^-^,
39	4.13	Scoulerine	C_19_H_21_NO_4_	327.1471	328.1553	0.1	296.1267[M+H-CH_3_OH]^+^, 178.0829[M+H-C_9_H_10_O_2_]^+^, 163.0598[M+H-C_9_H_10_O_2_-CH_3_]^+^,
40	4.16	Rutin*	C_27_H_30_O_16_	610.1534	609.1447	1.8	300.9992[M-H-Rutinose]^-^, 151.0054[A^1,3^]-
41	4.23	Isovitexin*	C_21_H_20_O_10_	432.1057	431.0988	0.6	341.0671[M-H-90]^-^, 311.0563[M-H-120]^-^
42	4.25	Tetrahydropalmatine	C_21_H_25_NO_4_	355.1783	356.1857	-0.6	341.1520[M+H-CH_3_]^+^, 192.0992[M+H-C_10_H_12_O_2_]^+^, 177.0754[M+H-C_10_H_12_O_2_-CH_3_]^+^, 149.1734[M+H-C_10_H_12_O_2_-CH_3_-CO]^+^ 165.0915[M+H-C_11_H_13_NO_2_]+,
43	4.26	Corlumine	C_21_H_21_NO_6_	383.1396	384.1447	0	368.1106[M+H-H_2_O]^+^, 249.0520
44	4.29	Chebulinic Acid*	C_41_H_32_O_27_	956.1631	955.1034	0.2	803.2881[M-H-galloyl]^-^, 651.3185[M-H-2galloyl]^-^, 633.1791[M-H-2galloyl-gallic]^-^, 499.4882[M-H-3galloyl]^-^
45	4.37	3-galloyl-2,4-chebuloyl-1,6-HHDP-glucose	C_41_H_30_O_27_	954.0975	953.0885	-0.2	454.2341[M-H-COOH]^2-^, 300.9994[Hexahydroxy diphenoyl-H]^-^,
46	4.38	Vitexin 2’’-O-rhamnoside	C_27_H_30_O_14_	578.1636	577.1554	-0.3	457.1158[M-H-120]^-^, 431.0808[M-H-Rha]^-^, 413.0879[M-H-Rha-H_2_O]^-^, 341.3668[M-H-Rha-90]^-^, 311.0567[M-H-Rha-120]^-^,
47	4.42	1,2,3,4,6-Pentagalloylglucose	C_41_H_32_O_26_	940.1182	939.1083	-2.1	469.0504[M-2H]^2-^, 787.0979[M-H-galloyl]^-^, 635.2447[M-H-2galloyl]^-^, 483.3546[M-H-3galloyl]^-^, 331.4498[M-H-4galloyl]^-^
48	4.49	(+)-Bicuculline*	C_20_H_17_NO_6_	367.1056	368.1145	1.1	307.0586[M+H-NH_2_CH_3_-H_2_O-CH_3_]^+^, 277.0499, 249.0520, 190.0849
49	4.65	Sylvestroside I	C_33_H_48_O_19_	748.279	747.2738	2.6	585.2177[M-H-glc]^-^, 423.1231[M-H-glc-glc]-
50	4.65	Isochlorogenic acid A	C_25_H_24_O_12_	516.1268	515.1195	0.8	353.0879[M-H-caffeoyl]^-^, 191.0553[M-H-2caffeoyl]^-^, 179.0343[M-H-2caffeoyl-H_2_O]^-^, 135.0446[caffeic acid-H-CO_2_]^-^
51	4.68	Isochlorogenic acid B	C_25_H_24_O_12_	516.1268	515.1195	0.8	353.0879[M-H-caffeoyl]^-^, 191.0553[M-H-2caffeoyl]^-^, 179.0343[M-H-2caffeoyl-H_2_O]^-^, 135.0446[caffeic acid-H-CO_2_]^-^
52	4.86	(-)-Bicuculline	C_20_H_17_NO_6_	367.1056	368.1145	1.1	307.0586[M+H-NH_2_CH_3_-H_2_O-CH_3_]^+^, 277.0499, 249.0520, 190.0849
53	5.01	Cassiaside B2	C_39_H_52_O_25_	920.2798	919.2737	-1.7	271.0601[M-H-4glc]^-^, 257.0369[M-H-4glc-CH_3_]^-^
54	5.09	Protopine	C_20_H_19_NO_5_	353.1263	354.1339	-0.3	336.1216[M+H-H_2_O]^+^, 188.0672[M+H-C_9_H_8_O_2_-H_2_O]^+^, 149,0582[M+H-C_11_H_11_NO_3_]^+^,
55	5.15	Cantleyoside	C_33_H_46_O_19_	746.2633	745.2544	-1.5	583.2032[M-H-glc]^-^, 421.1159[M-H-2glc]^-^
56	5.21	Crocin I*	C_44_H_64_O_24_	976.3787	975.3721	1.3	813.3179[M-H-glc]^-^, 651.2274[M-H-2glc]^-^
57	5.26	Medicarpin	C_16_H_14_O_4_	270.0892	269.0831	-0.2	159.0242[M-H-C_6_H_6_O_2_]^-^
58	5.28	Cassiaside	C_20_H_20_O_10_	404.4257	403.1398	-0.4	257.0454[M-H-glc]^-^
59	5.36	Aurantio-obtusin-6-O-β-D-glucoside	C_23_H_24_O_12_	492.1268	491.1199	-1.4	476.0960[M-H-CH_3_]^-^, 461.0760[M-H-2CH_3_]^-^, 329.0694[M-H-glc]^-^, 314.0418[M-H-glc-CH_3_]^-^, 286.0182[M-H-glc-CH_3_-CO]^-^, 258.0227[M-H-glc-CH3-2CO]^-^
60	5.37	Chelidonine	C_20_H_19_NO_5_	353.1263	354.1339	-0.3	336.1216[M+H-H_2_O]^+^, 322.1068[M+H-NH_2_CH_3_]^+^,
61	5.39	Coptisine	C_19_H_14_NO_4_+	320.0923	320.0899	2.4	292.0939[M-CO]^+^, 262.0863[M-CH_2_O]^+^
62	5.46	Diosbulbin J- glucoside	C_26_H_36_O_12_	540.1843	539.1772	-0.5	377.1246[M-H-glc]^-^, 349.0827[M-H-glc-CO]^-^
63	5.51	Jatrorrhizine	C_20_H_20_NO_4_+	338.1392	338.1395	0.5	323.1228[M-CH_3_]^+^, 308.0904[M-2CH_3_]^+^, 280.0947[M-2CH_3_-CO]^+^,
64	5.63	Crocin II	C_38_H_54_O_19_	814.3259	813.3167	-1.4	651.2267[M-H-glc]^-^, 489.2274[M-H-2glc]^-^
65	5.77	Apigenin	C_15_H_10_O_5_	270.0892	269.0831	-0.4	251.0343[M-H-H_2_O]^-^, 241.4335[M-H-CO]^-^
66	6.04	Quercetin*	C_15_H_10_O_7_	302.0427	301.0364	-0.4	283.1690[M-H-H_2_O]^-^, 273.0665[M-H-glc-CO]^-^, 163.0189[A^0,2^]^-^, 151.0033[A^1,3^]^-^
67	6.17	Diosbulbin L- glucoside	C_25_H_32_O_12_	524.1894	523.1837	2.1	361.2174[M-H-glc]^-^, 333.0882[M-H-glc-CO]^-^
68	6.34	Obtusin	C_18_H_16_O_7_	344.0896	343.0824	-0.7	328.0227[M-H-CH_3_]^-^,313.0284[M-H-2CH_3_]^-^, 285.0477[M-H_2_CH_3_-CO]^-^, 270.9883[M-H-3CH_3_-CO]-, 242.9933[M-H-3CH_3_-2CO]
**69**	6.37	Dihydrochelerythrine	C_21_H_19_NO_4_	349.1314	350.136	-3.2	334.1060[M+H-CH_3_]^+^,
70	6.87	Dehydrocorydaline	C_22_H_24_NO_4_+	366.1705	366.1695	-1	366.1695[M]^+^, 351.1484[M-CH_3_]^+^, 334.1060[M-2CH_3_]^+^,
71	7.10	Kaempferol	C_15_H_10_O_6_	286.0477	285.0397	-0.2	257.0296[M-H-CO]^-^, 243.0245[M-H-C_2_H_2_O]^-^, 227.0345[M-H-CO-CHOH]^-^, 151.0076[A^1,3^-H]^-^, 133.0318 [B^1,3^]^-^
72	7.46	Formononetin	C_16_H_12_O_4_	268.2604	267.0657	0	252.0417[M-H-CH_3_]^-^
73	7.68	Crocin I/Cis-trans isomer	C_44_H_64_O_24_	976.3787	975.3695	1.3	813.3179[M-H-glc]^-^, 651.2274[M-H-2glc]^-^
74	7.99	Cis- Crocin II/Cis-trans isomer	C_38_H_54_O_19_	814.3259	813.3153	-1.4	651.2267[M-H-glc]^-^, 489.2274[M-H-2glc]^-^
75	8.13	Aurantio-obtusin	C_17_H_14_O_7_	330.0739	329.0667	-1.4	314.0415[M-H-CH_3_]^-^, 299.0197[M-H-2CH_3_]^-^,285.0402[M-H-CO_2_]^-^, 271.0520[M-H-2CH_3_-CO]^-^
76	8.25	Trihydroxy-α-boswellic acid	C_30_H_48_O_6_	504.3451	503.3388	1.6	-
77	8.30	Trihydroxy-β-Boswellic acid	C_30_H_48_O_6_	504.3451	503.3388	1.6	-
78	8.43	Dihydroxy-11-Keto-β-boswellic acid	C_30_H_46_O_6_	502.3294	501.3236	2	-
79	8.55	Isorhamnetin	C_16_H_12_O_7_	316.0583	315.0514	1.3	300.0275[M-H-CH_3_]^-^, 272.0320[M-H-CH_3_-CO]^-^, 151.0469[A^1,3^]^-^, 163.0424[B^1,3^]^-^
80	8.60	Taurochenodeoxycholic acid (TDCA)	C_26_H_45_NO_6_S	499.2968	498.2893	-1.1	480.3216[M-H-H_2_O]^-^, 427.3157[M-H-HSO_3_]^-^, 373.2737[M-H-HSO_3_-C_2_H_5_N]^-^
81	9.21	Cholic Acid (CA)	C_24_H_40_O_5_	408.2876	407.2807	-1.6	389.2747[M-H-H_2_O]^-^, 362.2839[M-H-COOH]^-^, 343.2640[M-H-COOH-H_2_O]^-^,
82	9.28	Kaempferide	C_16_H_12_O_6_	300.0634	299.0562	0.3	284.0323[M-H-CH_3_]^-^, 271.0606[M-H-CO]^-^, 151.0076[A^1,3^-H]^-^
83	9.56	Ursodeoxycholic acid*(UDCA)	C_24_H_40_O_4_	392.2927	391.2856	0.3	373.2657[M-H-H_2_O]^-^, 346.0562[M-H-COOH]^-^, 328.0393[M-H-COOH-H_2_O]^-^,
84	9.77	Dihydroxy-α-boswellic acid	C_30_H_48_O_5_	488.3502	487.3443	1.7	-
85	10.05	Dihydroxy-β-boswellic acid	C_30_H_48_O_5_	488.3502	487.3443	1.7	-
86	10.20	Galangin	C_15_H_10_O_5_	270.0528	269.0454	0.3	251.1652[M-H-H_2_O]-, 225.0555[M-H-CO_2_]-, 151.1026[A^1,3^]-
87	10.33	Rhamnazin	C_17_H_14_O_7_	330.074	329.0668	0.5	314.0431[M-H-CH_3_]^-^, 299.0197[M-H-CH_3_-CH_3_]^-^, 271.02487[M-H-2CH_3_-CO]^-^, 165.1026[A^1,3^]^-^
88	11.35	11-hydroxy-α-boswellic acid	C_30_H_48_O_4_	472.3553	471.3478	2	409.2831[M-H-H_2_O-CO_2_]^-^, 239.2327[B^8,9^]^-^, 233.2012[D^8,9^]^-^
89	11.38	11-hydroxy-β-boswellic acid	C_30_H_48_O_4_	472.3553	471.3476	2	409.2831[M-H-H_2_O-CO_2_]^-^, 239.2327[B^8,9^]^-^, 233.2012[D^8,9^]^-^
90	11.48	2α-hydroxy-oleanolic acid	C_30_H_48_O_4_	472.3553	471.3478	2	453.3402 [M-H-H_2_O]^-^, 407.3328 [M-H-H_2_O-CO_2_]^-^,
91	12.78	Oleanolic acid*	C_30_H_48_O_3_	456.3604	455.3528	-1.8	393.3420[M-H-H_2_O-CO_2_]^-^, 239.1201[B^8,9^]^-^, 217.2012[D^8,9^]^-^
92	12.87	α-boswellic acid	C_30_H_48_O_3_	456.3604	455.3532	-1.8	393.3420[M-H-H_2_O-CO_2_]^-^, 239.1201[B^8,9^]^-^, 217.2012[D^8,9^]^-^
93	13.21	Myristic acid	C_14_H_28_O_2_		227.2017	0.1	-
94	12.89	Acetyl-11-keto-β-boswellic acid	C_32_H_48_O_5_	512.3502	511.3469	0.5	469.8469[M-H-C_2_H_2_O]^-^, 407.3417[M-H-C_2_H_2_O-H_2_O-CO_2_]^-^, 280.1628[B^8,9^]^-^, 231.8353[D^8,9^]^-^
95	13.82	3-Acetyl-β-boswellic acid	C_32_H_50_O_4_	498.3709	497.3632	1.6	455.2737[M-H-C_2_H_2_O]^-^, 393.8599[M-H-C_2_H_2_O-H_2_O-CO_2_]^-^, 280.8910[B^8,9^]^-^, 217.8976[D^8,9^]^-^
96	14.70	β-boswellic acid*	C_30_H_48_O_3_	456.3604	455.3532	-1.8	393.3420[M-H-H_2_O-CO_2_]^-^, 239.1201[B^8,9^]^-^, 217.2012[D^8,9^]^-^

* Components identified with standard substance.

### Network pharmacology analysis

#### Potential bioactive compounds and targets of ELP in the treatment of RA

**Fig 2:**
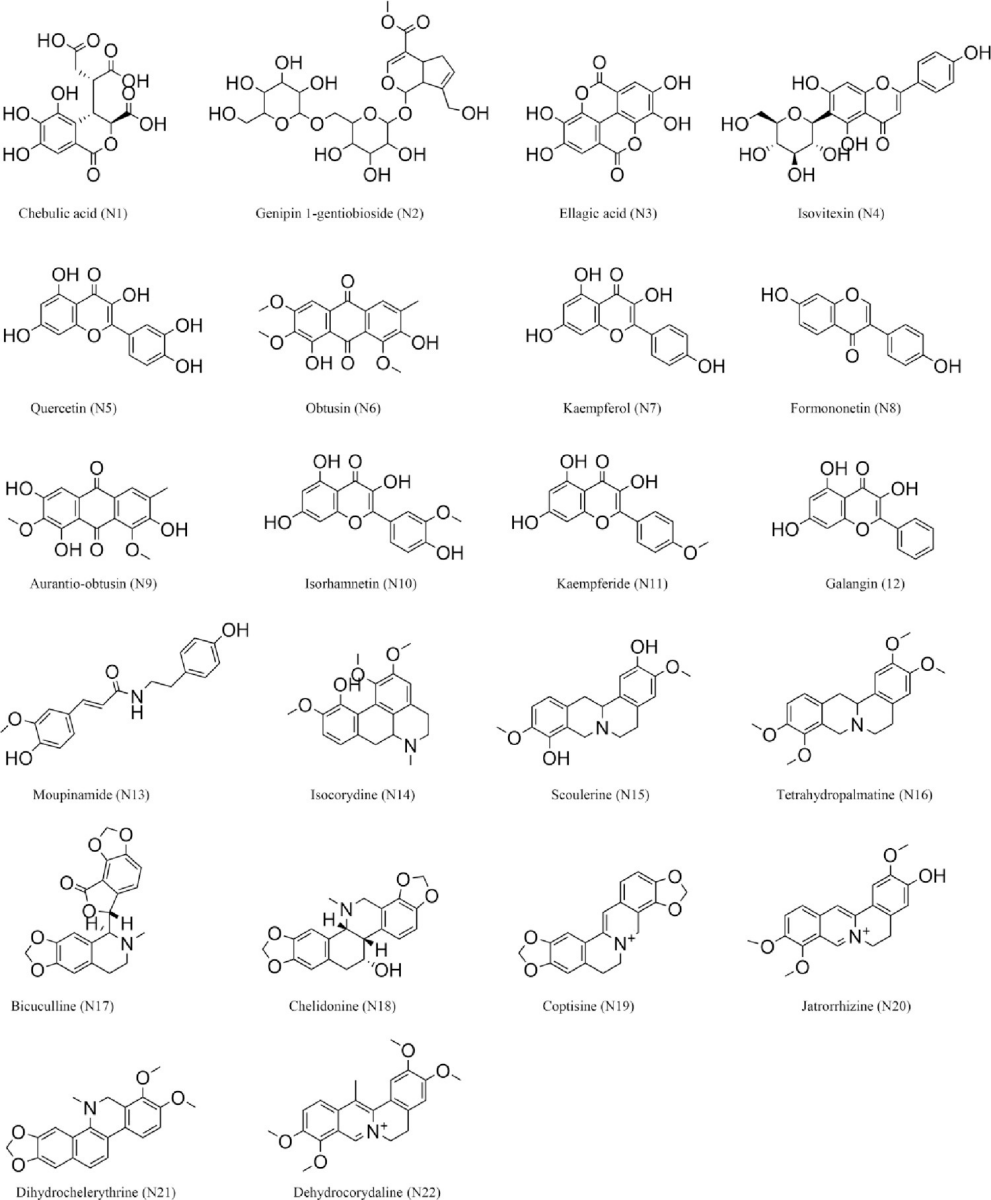
Structures of 22 potential bioactive compounds of ELP.

**Fig 3:**
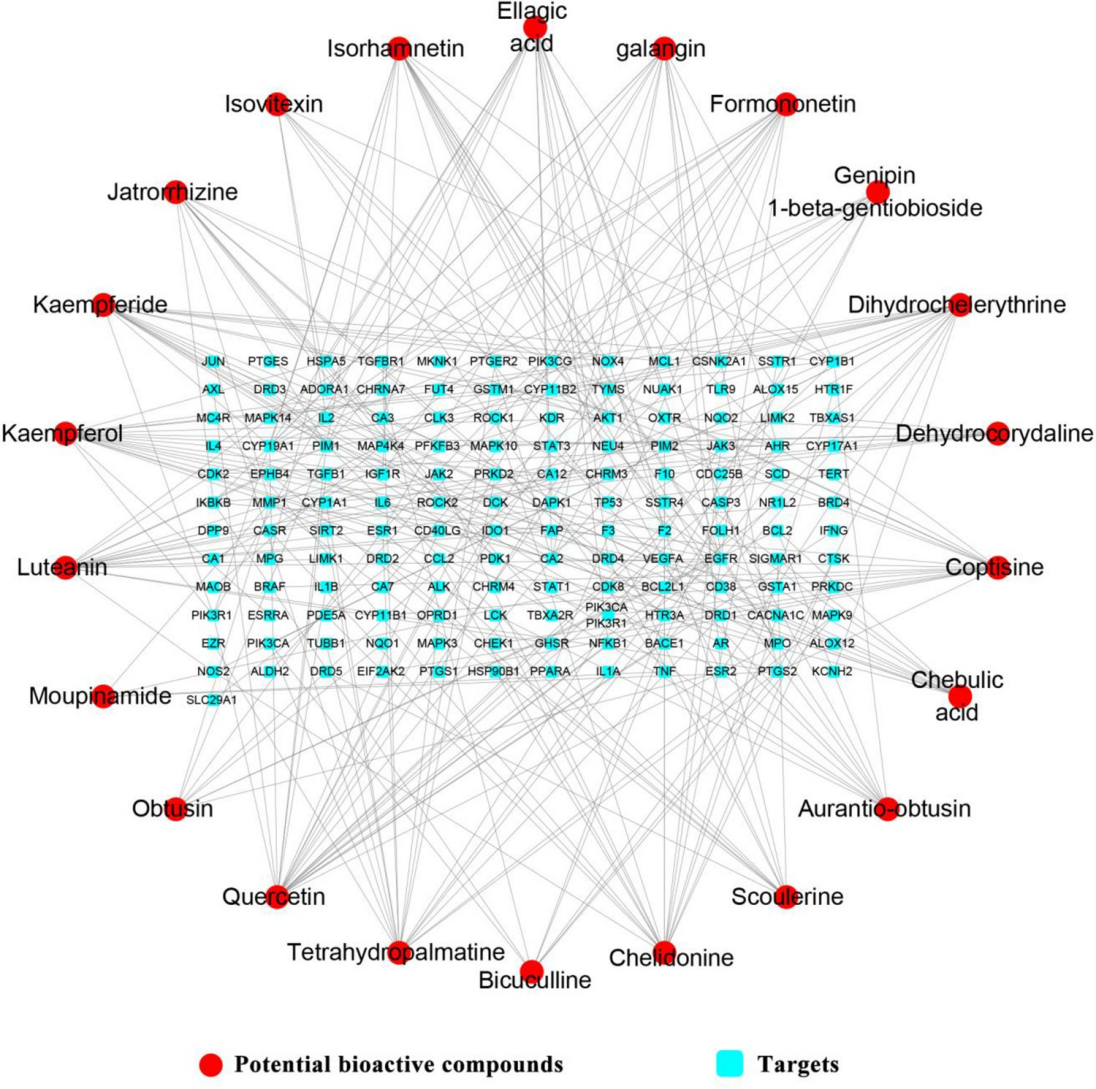
Compound-target network of ELP. In the network, there are 167 nodes and 292 edges. 22 potential bioactive compounds have interactions with 145 protein targets. The red circles represent the bioactive compounds, and the blue squares represent the targets.

**Fig 4:**
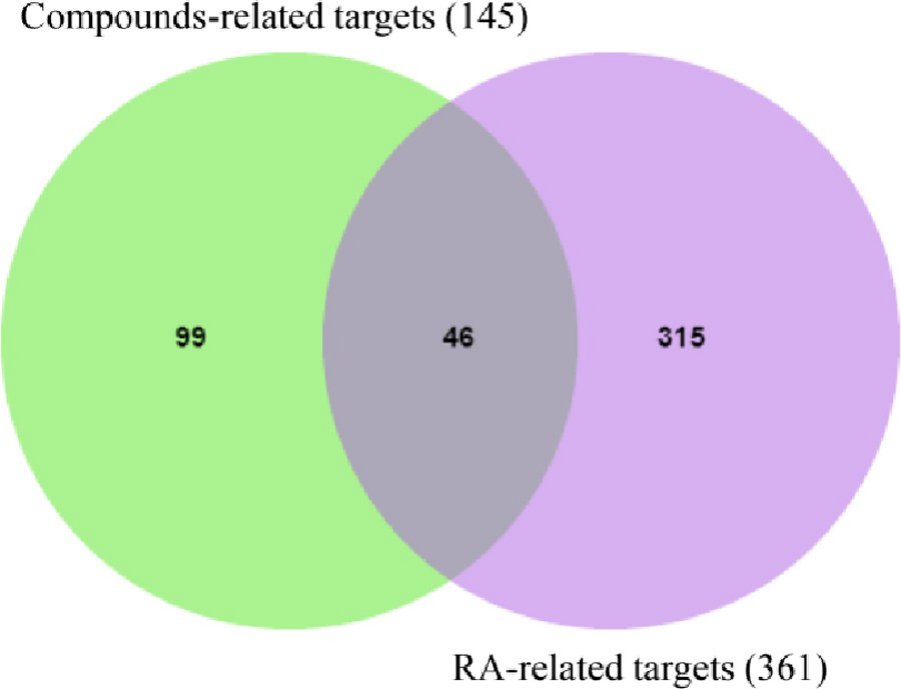
Venn diagram of the drugs and disease targets.

After ADME screening, 22 potential bioactive compounds (OB ≥ 30%, DL ≥ 0.18) in ELP were identified (Tables 2 and S2). Firstly, 145 potential targets of the 22 potential bioactive compounds were obtained from the Swiss Target Prediction platform (Figs 2 and 3) (S2 Table). Secondly, 361 RA-related targets were retrieved from GeneCards, CTD, and OMIM databases (S3 Table). Finally, 46 targets directly and indirectly associated with RA were obtained by precisely matching the potential targets of the above two steps through the online tool Venny 2.1 (http://bioinfogp.cnb.csic.es/tools/venny/) (Fig 4).

**Table 2:** Potential bioactive compounds ADME values of ELP (OB ≥ 30%, DL ≥ 0.18).

Number	Molecule ID	PubChem CID	Molecular name	OB (%)	DL
N1	MOL006826	71308174	chebulic acid	72.00	0.32
N2	MOL009038	3082301	Genipin 1-gentiobioside	45.58	0.83
N3	MOL001002	5281855	Ellagic acid	43.06	0.43
N4	MOL002322	162350	Isovitexin	31.29	0.72
N5	MOL000098	5280343	Quercetin	46.43	0.28
N6	MOL006475	155380	Obtusin	81.43	0.4
N7	MOL000422	5280863	Kaempferol	41.88	0.24
N8	MOL000392	5280378	Formononetin	69.67	0.21
N9	MOL006472	155011	Aurantio-obtusin	31.55	0.37
N10	MOL000354	5281654	Isorhamnetin	49.6	0.31
N11	MOL004564	5281666	Kaempferide	73.41	0.27
N12	MOL002563	5281616	Galangin	45.55	0.21
N13	MOL008647	5280537	Moupinamide	86.71	0.26
N14	MOL001467	10143	Luteanin	55.63	0.55
N15	MOL000217	439654	Scoulerine	32.28	0.54
N16	MOL004071	5417	Tetrahydropalmatine	73.94	0.64
N17	MOL000791	185838	Bicuculline	69.67	0.88
N18	MOL001481	978315	Chelidonine	48.32	0.86
N19	MOL001458	72322	Coptisine	30.67	0.86
N20	MOL006397	72323	Jatrorrhizine	30.44	0.75
N21	MOL001461	485077	Dihydrochelerythrine	32.73	0.81
N22	MOL004204	34781	Dehydrocorydaline	41.98	0.68

#### Protein-Protein Interaction (PPI) network

**Fig 5:**
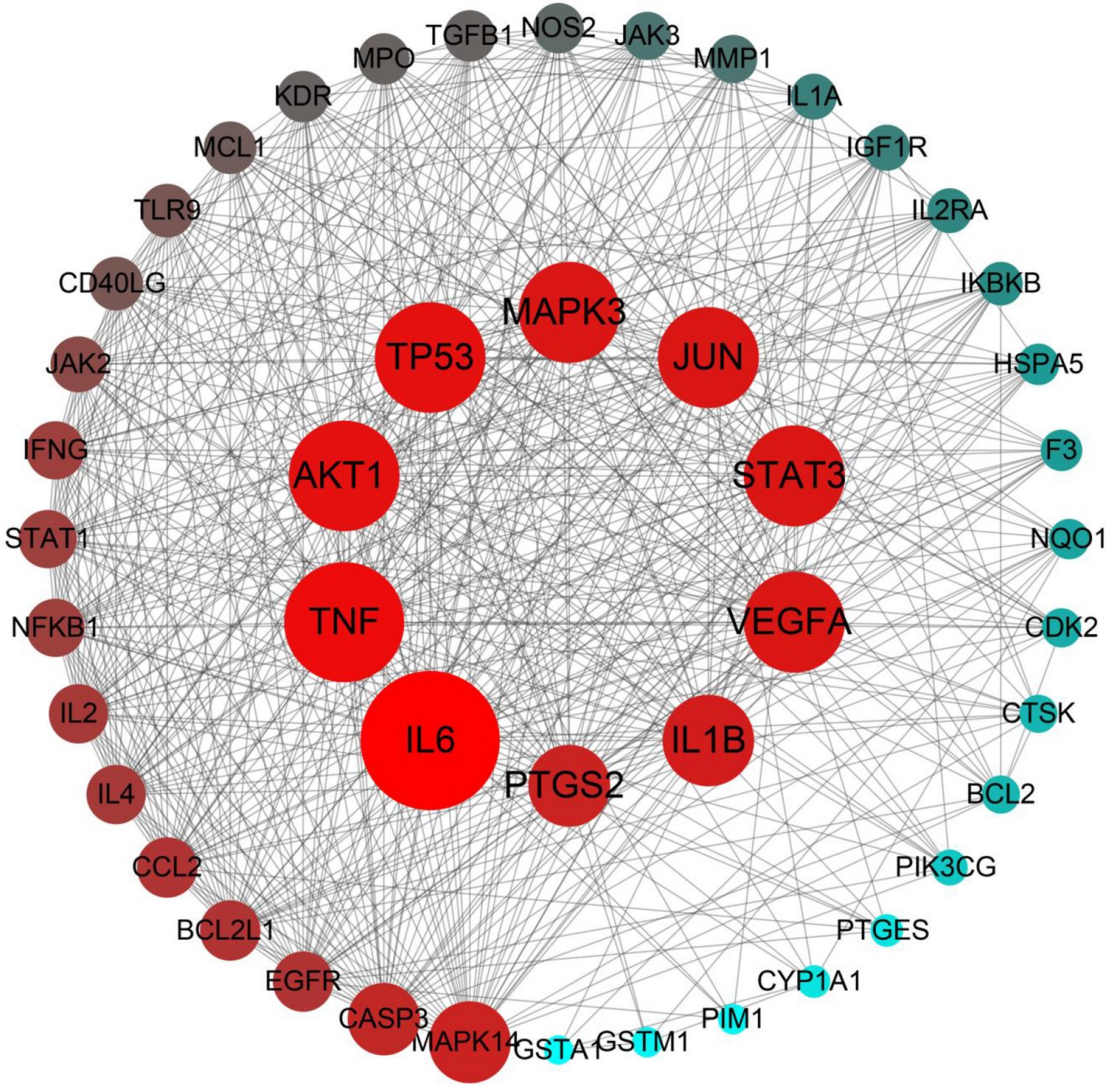
PPI network of ELP for the treatment of RA. In the network, there are 46 nodes and 563 edges. Each node represents the relevant gene, the size and color of the node represent the value of the free degree. (The larger the node, the redder the color, the greater the free degree).

The PPI relationship of 46 target genes was obtained by the STRING tool, and the visualization was realized by Cytoscape 3.8.0 software. The network of PPI relationships contained 46 nodes and 563 edges when a combined score of > 0.4 was used (Fig 5 and S4 Table). The 10 target genes with the highest connectivity degree were selected as the hub genes for RA. Thus, the hub genes, which might play a crucial role in RA progression, were IL6, TNF, TP53, AKT1, JUN, VEGFA, MAPK3, STAT3, IL1B, and PTGS2.

#### Gene Ontology (GO) function and KEGG pathway enrichment analysis

**Fig 6:**
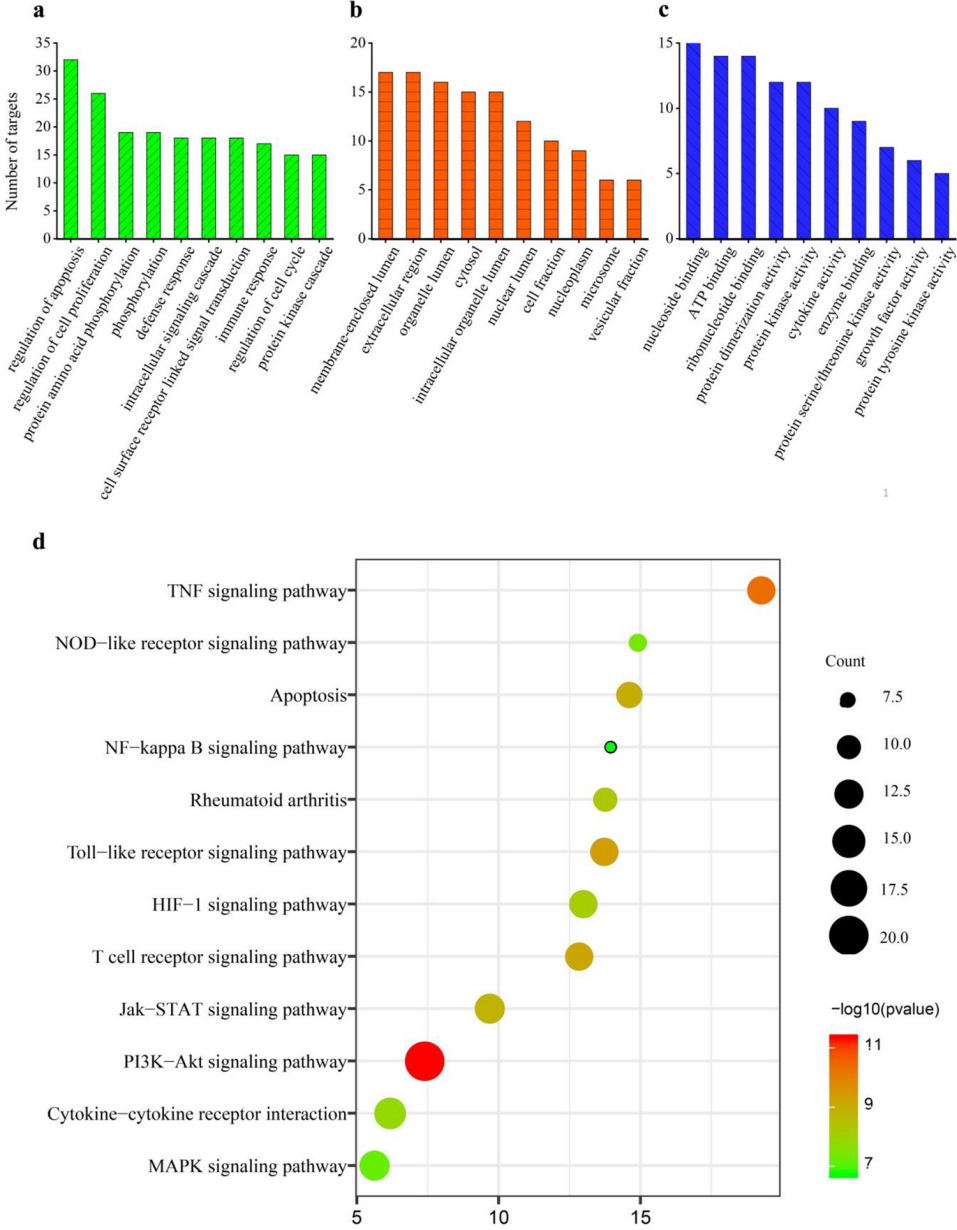
The results of GO enrichment and KEGG pathway analysis by DAVID. a: Biological process (BP), b: Cell component (CC), c: Molecular function (MF), d: KEGG pathway analysis. The *P*-value of each biological process was less than 0.01. In the bar plots, each bar represents a GO term on the vertical axis (Fig 6A–6C). The number of genes enriched in each term is represented on the vertical axis. The color of each bar represents each GO term. Similarly, in the bubble graphs, each bubble represents a KEGG path on the vertical axis (Fig 6D). The number of the genes is represented on the horizontal axis. The size of each bubble indicates the number of genes enriched in each KEGG pathway. The larger the bubble, the greater is the number of genes involved in the pathway. The color of each bubble represents the adjusted P-value for each KEGG path. The redder the bubble, the smaller the adjusted *P*-value is.

GO function and KEGG pathway enrichment analysis of the 46 candidate target genes were conducted by DAVID v6.8 to explore the molecular mechanism of ELP in treating RA. GO evaluations were illustrated using biological process (BP), cell component (CC), and molecular function (MF) terms (Fig 6A–6C). A total of 79 enrichment results in the related items of BP, involving regulation of apoptosis, protein amino acid phosphorylation, defense response, and intracellular signaling cascade; 20 enrichment results are related to CC, which includes a membrane-enclosed lumen, organelle lumen, cytosol, and cell fraction; 39 enrichment processes are related to the MF which cover the nucleoside binding, ATP binding, protein kinase activity, cytokine activity and so on. Enrichment results of each *p*-value were calculated (*p* < 0.01 was considered as significant enrichment). Subsequently, a total of 29 signaling pathways were obtained (*p* < 0.05), and 12 signaling pathways related to RA were identified (Table 3 and Fig 6D). Meanwhile, the top 6 signaling pathways closely associated with RA were screened according to the number of targets. Hence, ELP probably produces anti-RA effects by synergistically regulating many biological pathways, such as PI3K-Akt signaling pathway, Cytokine-cytokine receptor interaction, JAK-STAT signaling pathway, MAPK signaling pathway, TNF signaling pathway, and Toll-like receptor signaling pathway, and so on.

**Table 3:** The results of KEGG pathway analysis by DAVID.

KEGG ID	KEGG pathway	Targets	Count	PValue	FDR
hsa04151	PI3K-Akt signaling pathway	EGFR, PIK3CG, IL6, PTGS2, TP53, NFKB1, BCL2L1, STAT1, CDK2, TGFB1, MMP1, STAT3, AKT1, IGF1R, CASP3, JUN, BCL2, VEGFA, MAPK3, NOS2	20	2.1757E-13	2.39E-10
hsa04060	Cytokine-cytokine receptor interaction	EGFR, IL4, IL6, CCL2, IL2RA, TNF, TGFB1, KDR, CD40LG, VEGFA, IFNG, IL1B, IL1A, IL2	14	1.22292E-07	0.000134
hsa04630	Jak-STAT signaling pathway	PIK3CG, IL4, AKT1, IL6, IL2RA, IFNG, PIM1, JAK2, JAK3, BCL2L1, STAT1, STAT3, IL2	13	2.85231E-09	3.13E-06
hsa04010	MAPK signaling pathway	EGFR, TNF, TP53, NFKB1, TGFB1, AKT1, CASP3, MAPK14, JUN, MAPK3, IL1B, IKBKB, IL1A	13	1.22111E-06	0.00134
hsa04668	TNF signaling pathway	EGFR, PIK3CG, AKT1, MAPK3, VEGFA, TP53, NFKB1, BCL2L1, IKBKB, STAT1, STAT3, TGFB1	12	8.36687E-12	9.18E-09
hsa04620	Toll-like receptor signaling pathway	PIK3CG, AKT1, IL6, TNF, MAPK14, JUN, MAPK3, IL1B, NFKB1, IKBKB, STAT1, TLR9	12	3.71248E-10	4.07E-07
hsa04660	T cell receptor signaling pathway	PIK3CG, IL4, AKT1, TNF, CD40LG, MAPK14, JUN, MAPK3, IFNG, NFKB1, IKBKB, IL2	12	7.73345E-10	8.48E-07
hsa04066	HIF-1 signaling pathway	EGFR, PIK3CG, AKT1, IGF1R, BCL2, MAPK3, TP53, NFKB1, IKBKB, CDK2	12	3.54481E-08	3.89E-05
hsa04210	Apoptosis	PIK3CG, AKT1, CASP3, TNF, BCL2, TP53, IL1B, NFKB1, BCL2L1, IKBKB, IL1A	11	1.53564E-09	1.68E-06
hsa05323	Rheumatoid arthritis	PIK3CG, AKT1, PTGS2, BCL2, TP53, NFKB1, NOS2, BCL2L1, IKBKB, CDK2	10	2.11664E-08	2.32E-05
hsa04621	NOD-like receptor signaling pathway	IL6, TNF, CCL2, MAPK14, MAPK3, IL1B, NFKB1, IKBKB	8	6.4748E-07	0.00071
hsa04064	NF-kappa B signaling pathway	PIK3CG, AKT1, MAPK3, PIM1, NFKB1, IKBKB, STAT3	7	7.4395E-06	0.008161

#### Network construction and analysis

**Fig 7:**
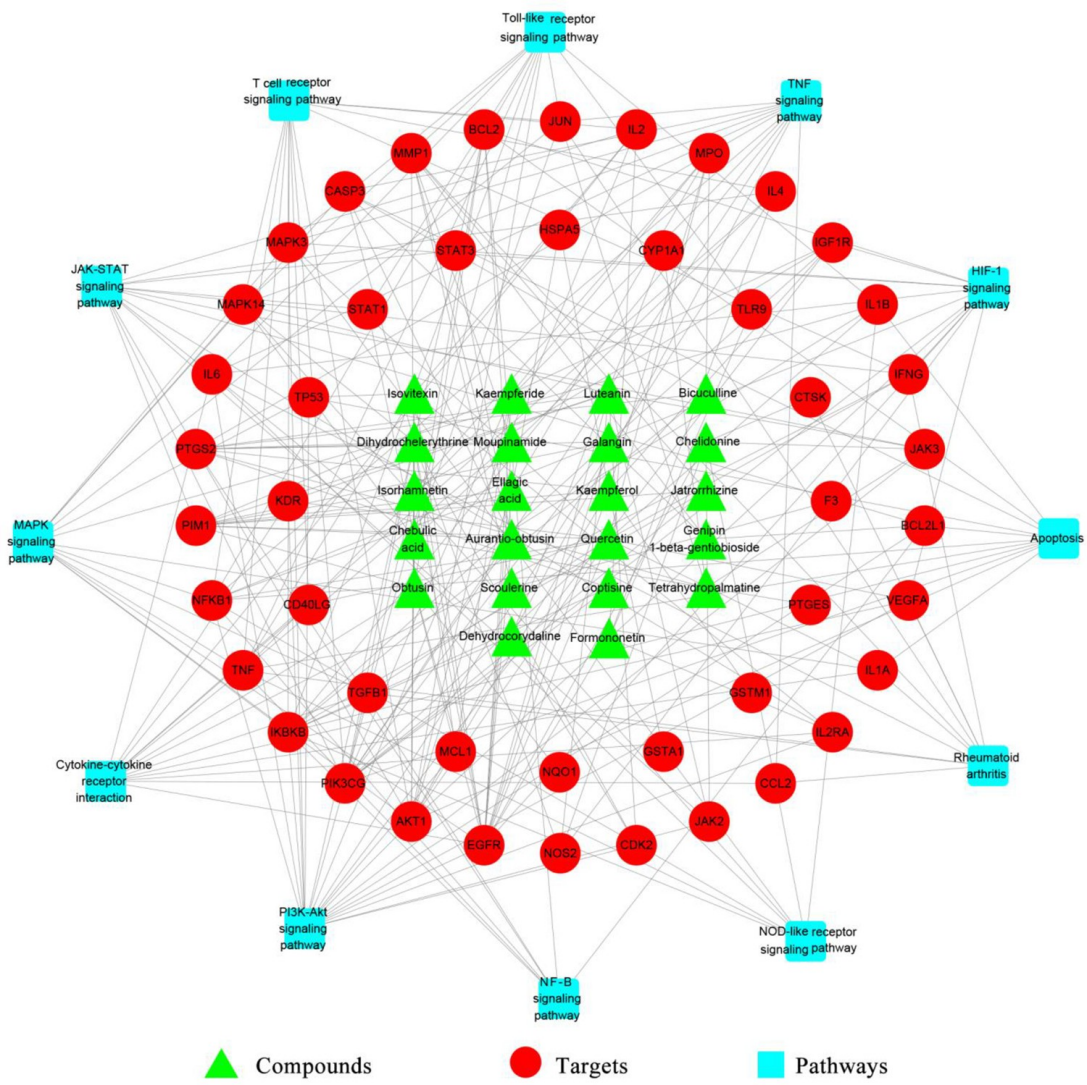
Compound-target-pathway Network (C-T-P) of ELP for treating RA. In the network, there are 80 nodes and 274 edges. The interaction relationship was shown between 22 potential bioactive compounds, 46 protein targets, and 12 signaling pathways. The green triangles represent the bioactive compounds, red circles represent targets, and blue squares represent signaling pathways.

A C-T-P Network of ELP for treating RA was constructed using Cytoscape 3.8.0 software (Fig 7). The network map showed that the relationships among 22 potential bioactive compounds, 46 protein targets, and 12 signaling pathways. With the excavation of the C-T-P network, the mechanism of ELP in treating RA was preliminarily understood.

### Molecular docking simulation

**Fig 8:**
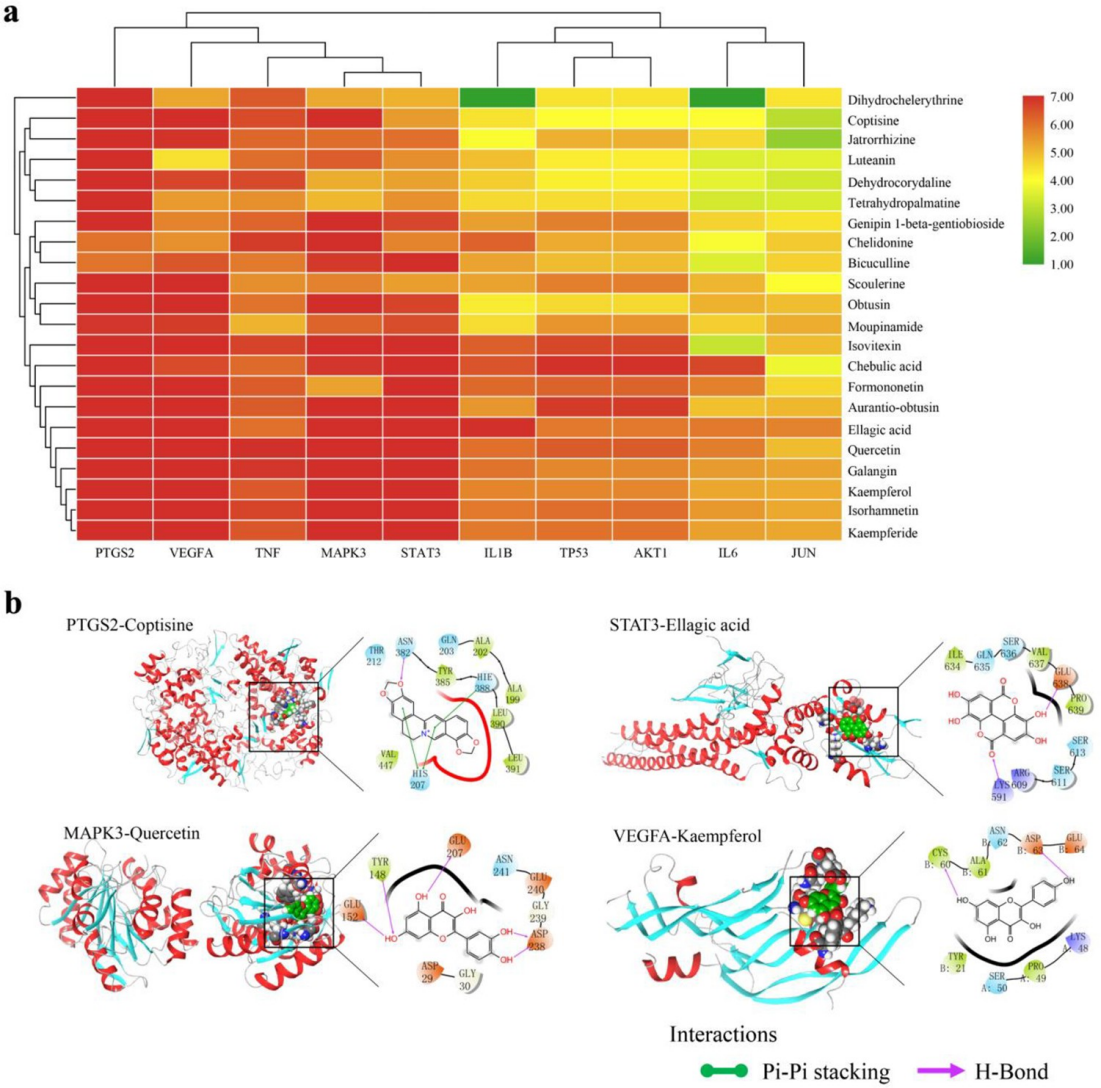
Molecular docking results between 10 targets and 22 potential bioactive compounds from ELP. a: Heatmap for docking score. The docking score represents a negative logarithm of the experimental dissociation/inhibition constant value (pKd/pKi). b: The typical Schematic representation of molecular docking (Including 3D and 2D structures). Droplet shapes represent amino acid groups that interact with compounds in 3Å. Capital letters represent the abbreviation for amino acids. (Color figures can be accessed in the online version).

Molecular docking simulation was performed using Maestro11.5 (Schrodinger Suite) between the 22 potential bioactive compounds and 10 key targets. The three-dimensional (3D) structures of the 10 selected targets were obtained from the PDB database (https://www.rcsb.org/), which is an archive that includes experimentally determined atomic-level 3D structures of biological macromolecules (DNA, RNA, and proteins) [33]. The docking scores were depicted in Fig 8A and S5 Table. The hydrogen bonding and π-π stacking were involved between the targets and the potential compounds. Finally, according to the heat map analysis, good molecular docking scores were highlighted between five promising bioactive compounds (ellagic acid, quercetin, kaempferol, galangin, coptisine) and five core targets (PTGS2, STAT3, VEGFA, MAPK3, TNF). The typical schematic representation of 3D and 2D molecular docking patterns of target proteins and compounds are shown in Fig 8B, including PTGS2 with coptisine, STAT3 with ellagic acid, MAPK3 with quercetin, and VEGFA with kaempferol. For instance, the binding mode of coptisine in the active site of PTGS2 has been represented in its 3D and 2D modes. Coptisine showed an H-bond interaction and three π-π stacking. The oxygen of coptisine forms a hydrogen bond with ASN382, and three π-π stacking were formed by binding the six-membered ring to HIE388 and HIS207, respectively.

## Discussion

As a common systemic inflammatory autoimmune disease, with high disability and incidence, RA can severely impair physical function and quality of life [34]. Recently, discoveries have improved the understanding of rheumatoid inflammation and its consequences [35]. The pathophysiology of RA involves chronic inflammation of the synovial membrane, which can destroy articular cartilage and juxta-articular bone [36]. RA is characterized by infiltration of the synovial membrane in joints with T cells, B cells, and monocytes, and the common symptoms are musculoskeletal pain, swelling, and stiffness in clinical practice [37]. As reported that the innate immune response produces some cytokines, among those, IL-1, IL-6, and TNF-α played important parts in the progress of RA [38]. In addition, the process of RA involves the activation of multiple inflammatory signaling pathways and interactions with inflammatory cytokines [39]. Nevertheless, given the uncertainty and complexity of its pathogenesis, there’s no specific medicine that can effectively treat RA. Modern therapies such as nonsteroidal anti-inflammatory drugs or pain medications only improve symptoms but do not prevent damage progression and irreversible disability [37]. The mainly therapeutic strategy is applicated disease-modifying antirheumatic drugs for patients with RA at present. For example, methotrexate, one of the therapeutic drugs, is the most effective and commonly used first-line drug [40]. Unsatisfactory, it has large side effects. Consequently, it is urgent to discover and develop a safe and effective therapeutic.

ELP, a traditional Tibetan patented prescription medicine, is one of the commonly used drugs to cure RA in clinical trials [2]. More importantly, according to the *Pharmacopoeia of the People’s Republic of China (2015)*, only *Pterocephalus hookeri* (C.B.Clarke) Hoeck has minor toxicity in ELP, and the clinical dosage is 1–3 g/d. In contrast, according to the proportion of 25 herbs in ELP, the dosage of *Pterocephalus hookeri* (C.B.Clarke)Hoeck is only 0.11 g/d, which is a safe dose. Therefore, ELP can be considered nontoxic. Hence, for the first time, a system pharmacological approach using UPLC-Q-TOF/MS and network pharmacology with molecular docking simulation was applied in this study to the prediction of promising bioactive compounds and mechanisms for the treatment of RA. In this work, it is found that ELP probably performed anti-RA effects via synergistically regulating many biological pathways, important six such as PI3K-Akt signaling pathway, Cytokine-cytokine receptor interaction, JAK-STAT signaling pathway, MAPK signaling pathway, TNF signaling pathway, and Toll-like receptor signaling pathway. Concurrently, good molecular docking scores were highlighted between five promising bioactive compounds (ellagic acid, quercetin, kaempferol, galangin, coptisine) and five core targets (PTGS2, STAT3, VEGFA, MAPK3, TNF).

Previous studies have suggested that the PI3K/Akt signaling pathway contributes to excessive cell proliferation, migration, and invasion in RA fibroblast-like synoviocytes (RA-FLSs) [41,42]. Excessive proliferation of FLSs is one of the critical features of RA, leading to cartilage and bone destruction [43]. Bone marrow MSCs, which play a key role in the healing of bone defects, have been applied for the treatment of RA via activation of the PI3K/AKT signaling pathway [44]. Consequently, according to the results of network analysis, one of the ELP mechanisms in anti-RA effects may be associated with regulating the PI3K/AKT signaling pathway. Cytokine-cytokine receptor interaction plays a vital role in both innate and adaptive inflammatory host defenses and development and repair processes aimed at the restoration of homeostasis, which is involved in the pathogenesis of inflammatory and autoimmune diseases including RA [45]. Notably, there are implicated in plenty of studies also suggesting cytokine-cytokine interactions involved in the pathogenesis of RA [46]. In addition, large postinjury increases in RA-associated markers and differential upregulation of the cytokine-cytokine receptor interaction pathway that is closely associated with inflammation [47]. JAK-STAT signaling pathway is a key player in RA progression [48]. In previous studies, the levels of serum cytokines TNF-α and IL-1β are increased in RA patients. Meanwhile, activation of the JAK2-STAT3 pathway is regulated by inflammatory cytokine stimulation during the progression of RA [49,50]. Accumulating studies indicated that the MAPK signal transduction pathway can regulate the inflammatory cytokine and downstream cell transduction pathways, thereby affecting the inflammation and destruction of joints [51]. The MAPK family includes p38MAPK, extracellular signal-regulated kinase (ERK), and c-Jun N-terminal kinase (JNK), which were activated in the synovium of RA patients [52,53]. It was reported that CCR5 silencing suppresses the inflammatory response, inhibits viability, and promotes apoptosis of synovial cells in RA rats by inhibiting the MAPK pathway [54]. TNF-a is a major cytokine implicated in RA [55]. The previous article revealed that miR-17 overexpression inhibited TRAF2 expression and its association with cIAP2, thereby suppressing the TNF-a signaling pathway and downstream inflammatory proteins in RA SFs [56]. Emerging evidence shows that the activation of the Toll-like receptor signaling pathway can initiate the perpetual cycle of inflammation in the arterial wall and joint synovium in patients with RA [57]. It is demonstrated that functional suppression of RGS1 inhibits the inflammatory response and angiogenesis by inactivating the TLR signaling pathway in rats with CIA [58]. These investigations improved the prediction of ELP against RA via inflammation associated with this pathway. However, it is necessary to verify them through further experimental research.

Literature for understanding the development of inflammatory arthritis revealed that LL-37 and IL17A can significantly enhance PTGS2 and TNF gene expression, then release its downstream pro-inflammatory cytokines, PGE2 and TNF, contributing to the enhancement of the pathogenesis mechanisms of inflammatory arthritis [59]. Meanwhile, PTGS2, IL1β, IL6, TNFA, and CCL20 have been shown that mediators transformed RA-FLS to be a major source of pro-inflammatory in the pathology of RA [60]. The active components of ELP inhibit the activation of RA-related signaling pathways by binding to PTGS2. Including NF-Kappa B signaling Pathway, VEGF signaling pathway, TNF signaling pathway, etc (S3B and S3D Fig). The STAT proteins are usually inactive cytoplasmic proteins. Excess continuous exposure of STAT proteins induced proinflammatory cytokines or growth factors causes the development of RA [61]. STAT3, one of the STAT family members, has been proposed as an early pathophysiological event in RA. It was demonstrated that three STAT3-regulated genes, BCL-2, SOCS3, and PIM1, could induce pathological lesions in RA via altered T cell effector function [62]. Key compounds may be involved in inhibiting STAT3 expression, thereby blocking the expression of downstream inflammatory factors involved in RA pathogenesis, and thereby blocking the immune response induced by the JAK-STAT signaling pathway (S3A Fig). VEGF is a multifunctional cytokine that expresses in macrophages and neutrophils, inducing leukocyte accumulation and collagen deposition [63]. There is evidence indicating that VEGFA promotes the migration and proliferation of endothelial cells, as well as inducing vascular permeability and mediating inflammation. VEGFA is a key factor in the development of pannus in RA [64]. Consequently, VEGFA inhibitors could disrupt new vessels and inhibit the delivery of nutritional proteins to sites of inflammation in RA [65]. VEGFA is the upstream cytokines of the VEGF signaling Pathway, PI3K-Akt signaling pathway, and other signaling pathways. Combining with VEGFA, the core compounds in ELP can inhibit the activation of the VEGF signaling pathway and PI3K-Akt signaling pathway, thus inhibiting the pathogenesis of RA (S3B Fig). Mitogen-activated protein kinases (MAPKs) are closely correlated with inflammatory diseases. MAPK3, one of the MAPKs, has the same function participating in the MAPK signaling pathway in RA [66]. The combination of core compounds in ELP with MAPK3 can inhibit the phosphorylation of MAPK3, and then block the RA immune response caused by MAPK Signaling Pathway (S3C Fig). Genes in the TNF family have been associated with RA and may be a potential therapeutic target [67]. Plenty of shreds of evidence illustrated that TNF perpetuates synovial inflammation via activating RA-FLS inducing a constellation of genes [55]. TNF is a key upstream target of the TNF signaling pathway, and the combination of the core compounds in ELP with TNF can block the RA immune response induced by TNF signaling Pathway (S3D Fig). According to the relationship between the above targets and signal pathways, it can be known that ELP exerts anti-RA effects through multiple components, multiple targets, and multiple pathways at the molecular level. Those provide strong evidence for the prediction of ELP to treat RA via intervention between these key targets, but it is necessary to further validate the claims using molecular biological methods.

It is necessary to clarify the activity ingredients of ELP revealing the scientific connotation of its on RA. Molecular docking analysis showed that five promising bioactive compounds, ellagic acid, quercetin, kaempferol, galangin, and coptisine, docking own stable. The above compounds mainly belong to polyphenols, flavonoids, and alkaloids, and a large investigation explicated that all these compounds are efficient in RA [68–70]. In addition, the contents of five bioactive compounds were studied. By comparing the UPLC-Q-TOF-MS total ion chromatogram of ELP, it was found that ellagic acid, Quercetin, Kaempferol, Galangin and Coptisine were the compounds with high content in 96 identified components. In particular, Ellagic acid, Quercetin, galangin. Specifically, Ellagic acid is a phenolic acid compound, which comes from *Phyllanthus emblica*, *Terminalia billerica* and *Terminalia chebula* in ELP and is the main compound of the above three herbs [71–73]. Quercetin, Kaempferol and Galangin are flavonoids, which widely exist in nature. The above three bioactive ingredients are contained in a large amount in the herbal medicine of ELP, such as *Cassia obtusifolia*, *Gossampinus malabarica*, *Abelmoschus manihot*, *Pterocephalus hookeri*, *Gentiana manshurica* and *Fraxinus rhynchophylla* [74]. Coptisine is an alkaloid component and an important compound of *Adhatoda vasica*. Previous studies have shown that ellagic acid alleviated the adjuvant-induced arthritis model in mice by modulation of pro-and anti-inflammatory cytokines (IL-1β, TNF-α, IL-17, IL-10, and IFN-γ) [75]. Quercetin could diminish myeloperoxidase activity and ROS levels to aid the control of autoimmune inflammation in patients with RA [5]. Kaempferol inhibits the migration and invasion of fibroblast-like synovial cells in RA by blocking the activation of the MAPK pathway [76]. Experiments showed that galangin improved human RA FLS by inhibition of the NF‑κB/NLRP3 pathway activation [77]. Coptisine has been reported to possess anti-inflammatory activity that significantly inhibited the IL-1β-induced NF-kB activation in human RA chondrocytes [78]. All the above shreds of evidence proved that the prediction of bioactive compounds in ELP anti-RA is reasonable and reliable.

## Conclusions

In this study, it was the first time that an integrative strategy based on UPLC-Q-TOF/MS coupled with the UNIFI informatics platform was applied for chemical profile analysis of ELP. A sum of 96 compounds was identified or tentatively characterized from 70% methanol extraction of ELP by comparing retention times, mass spectra with authentic standards, fragmentation behaviors, and data previously reported, including tannins, flavonoids, penylpropanoids, terpenoids, quinonoids, steroids, alkaloids, and other compounds. Furthermore, based on the traditional Tibetan clinical efficacy and identified compounds of ELP in the treatment of RA, a C-T-P network was constructed by relying on the network pharmacology method. In addition, good molecular docking scores were highlighted between 5 promising bioactive compounds (ellagic acid, quercetin, kaempferol, galangin, coptisine) and 5 core targets (PTGS2, STAT3, VEGFA, MAPK3, TNF). These targets were further related to the key signaling pathways such as PI3K/Akt, JAK-STAT, MAPK, TNF, Cytokine-cytokine receptor interaction, and Toll-like receptor, which could explain the anti-RA effects of ELP.

In summary, a system pharmacological approach, using UPLC-Q-TOF/MS and network pharmacology with molecular docking simulation, was applied to the prediction of promising bioactive compounds in ELP and mechanisms for the treatment of RA, which provides a new idea for the research of other Tibetan medicine prescriptions. Nevertheless, the bioactive compounds, biological targets, and signaling pathways predicted the need to be confirmed and validated using the CIA rat model in further studies.

## Supporting information

S1 Fig Chemical structures of the compounds identified from ELP. (DOCX)

S2 Fig The fragmentation pathways of representative compounds from ELP. (DOCX)

S3 Fig KEGG enrichment diagram. (DOCX)

S1 Table The composition of ELP. (DOCX)

S2 Table Potential bioactive compounds ADME values of ELP. (DOCX)

S3 Table Targets of 22 potential bioactive compounds. (DOCX)

S4 Table 361 RA-related targets were retrieved from GeneCards, CTD, and OMIM databases. (DOCX)

S5 Table Target information of ELP in the treatment of RA. (DOCX)

S6 Table Results of a molecular docking simulation. (DOCX)

S7 Table Target information and native docking validation of TNF, VEGFA, MAPK3, STAT3 and PTGS2. (DOCX)

S1 Graphical abstract (TIF)
