# Supplementary material for: Elucidating the material basis and potential mechanisms of Ershiwuwei Lvxue Pill acting on rheumatoid arthritis by UPLC-Q-TOF/MS and network pharmacology
Source: PLoS One. 2022 Feb 7;17(2):e0262469. doi: 10.1371/journal.pone.0262469 (PMC8820630; doi:10.1371/journal.pone.0262469)
Supplement: S3 Fig — (DOCX) [file pone.0262469.s003.docx]

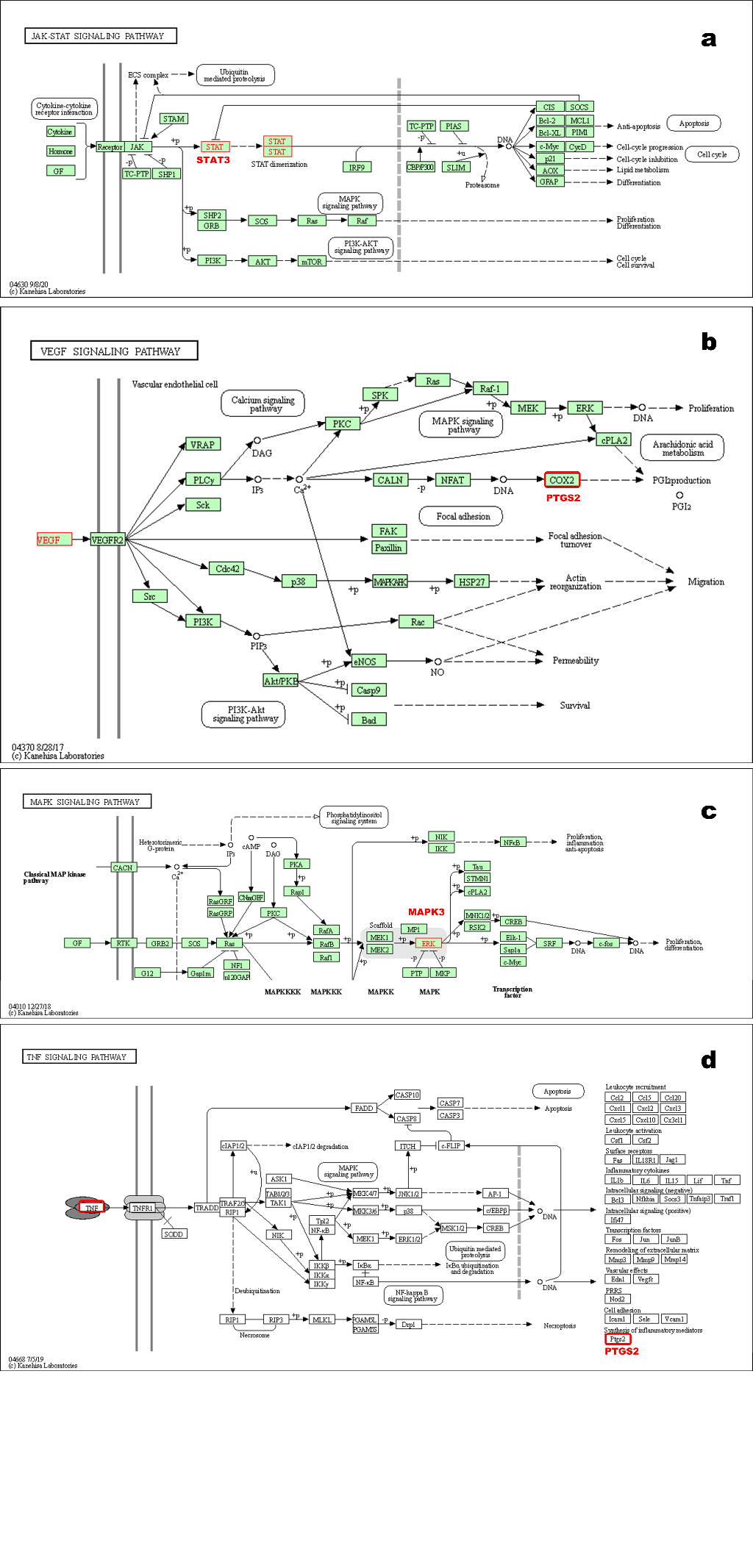


S3 Fig. KEGG enrichment diagram. (a) JAK-STAT signaling pathway. (b) VEGF signaling pathway. (c) MAPK Signaling Pathway. (d) TNF signaling pathway. pathway. Red asterisks represent core targets.
