# Supplementary material for: Elucidating the material basis and potential mechanisms of Ershiwuwei Lvxue Pill acting on rheumatoid arthritis by UPLC-Q-TOF/MS and network pharmacology
Source: PLoS One. 2022 Feb 7;17(2):e0262469. doi: 10.1371/journal.pone.0262469 (PMC8820630; doi:10.1371/journal.pone.0262469)
Supplement: S1 Table — (DOCX) [file pone.0262469.s004.docx]

S1 Table. The composition of ELP.

| No. | Pharmaceutical name | Botanical or zoological name | Family and plant part use | Chinese name | Content(g) |
| --- | --- | --- | --- | --- | --- |
| 1 | Asini sanguis pulvis | *Equus asinus* Linnaeus | Equidae; dry polypite | Lv xue | 50 |
| 2 | Iignum rhamnellae | *Rhamnella gilgitica* Mansf. et Melch | Rhamnaceae; dry polypite | Sheng deng gao | 30 |
| 3 | Dalbergiae odoriferae lignum | *Dalbergia odorifera* T.C.Chen | Leguminosae; heartwood | Jiang xiang | 80 |
| 4 | Santali albi lignum | *Santalum album* L | Santalaceae; heartwood | Tan xiang | 50 |
| 5 | Terminaliae belliricae fructus | *Terminalia bellirica* (Gaertn.) Roxb | Combretaceae; fruit | Mao he zi | 80 |
| 6 | Chebulae fructus | *Terminalia chebula* Retz | Combretaceae; fruit | He zi | 150 |
| 7 | Travertine | Calcareous tuff | Carbonate minerals | Shi hui hua | 100 |
| 8 | Phyllanthi fructus | *Phyllanthus emblica* L | Euphorbiaceae; fruit | Yu gan zi | 100 |
| 9 | Myristicae semen | *Myristica fragrans* Houtt | Myristicaceae; kernel | Rou dou kou | 30 |
| 10 | Caryophylli flos | *Eugenia caryophyllata* Thunb | Myrtaceae; bud | Ding xiang | 30 |
| 11 | Tsaoko fructus | *Amomum tsao-ko* Crevost & Lemarié | Zingiberaceae; fruit | Cao guo | 30 |
| 12 | Amomi fructus rotundus | *Amomum kravanh* Pierre ex Gagnep | Zingiberaceae; fruit | Dou kou | 30 |
| 13 | Cassiae semen | *Cassia obtusifolia* L | Leguminosae; seed | Jue ming zi | 50 |
| 14 | Olibanum | *Boswellia carteri* Birdw | Burseraceae; resin | Ru xiang | 50 |
| 15 | Gossampini flos | *Gossampinus malabarica* (DC.) Merr | Bombacaceae; flower | Mu mian hua | 30 |
| 16 | Ambrette seed | *Abelmoschus manihot* (L.) Medik | Malvaceae; seed | Huang kui zi | 50 |
| 17 | Pterocephali herba | *Pterocephalus hookeri* (C.B.Clarke) Hoeck | Dipsaceae; herba | Yi shou cao | 70 |
| 18 | Gentianae radix et rhizoma | *Gentiana manshurica* Kitag | Gentianaceae; root and rhizome | Long dan cao | 80 |
| 19 | Saxifrage | *Saxifraga pasumensis* Marg.et Shwa | Saxifragaceae; herba | Lian zuo hu er cao | 70 |
| 20 | Vasaka | *Adhatoda vasica* Nees | Acanthaceae; flower | Ba xia ga | 70 |
| 21 | Fallopia aubertii | *Tinospora sinensis* （Lour.）Merr | Menispermaceae; rattan | Kuan jin teng | 100 |
| 22 | Fraxini cortex | *Fraxinus rhynchophylla* Hance | Oleaceae; bark | Qin pi | 80 |
| 23 | Moschus | *Moschus berezovskii* Flerov | Cervidae; secreta | She xiang | 1 |
| 24 | Croci stigma | *Crocus sativus* L | Iridaceae; stigmas | Xi hong hua | 10 |
| 25 | Bovis calculus | *Bos taurus domesticus* Gmelin | Bovidae; gall-stone | Niu huang | 1 |

All the names are from The Pharmacopoeia of the People's Republic of China (2015 Edition).

The botanical names have been updated with www.theplantlist.org.

The content of 25 traditional medical herbs of ELP came from "Drug standards of the ministry of health in the People's Republic of China" Tibetan medicine volume 1995.
