# Supplementary material for: Elucidating the material basis and potential mechanisms of Ershiwuwei Lvxue Pill acting on rheumatoid arthritis by UPLC-Q-TOF/MS and network pharmacology
Source: PLoS One. 2022 Feb 7;17(2):e0262469. doi: 10.1371/journal.pone.0262469 (PMC8820630; doi:10.1371/journal.pone.0262469)
Supplement: S2 Table — (DOCX) [file pone.0262469.s005.docx]

S2 Table. Potential bioactive compounds ADME values of ELP.

| NO. | Molecular name | Water solubility | Oil-water partition coefficient(logP) | Plasma protein binding rate（PPB） | P-glycoproteinp-inhibitor | P450 enzyme metabolites | | | | | Drug half-life(HL) | Blood components(CL) |
| --- | --- | --- | --- | --- | --- | --- | --- | --- | --- | --- | --- | --- |
|  |  |  |  |  |  | CYP1A2 inhibitor | CYP2C19 inhibitor | CYP2C9 inhibitor | CYP2D6 inhibitor | CYP3A4 inhibitor |  |  |
| N1 | chebulic acid | almost insoluble | 0.591 | 81.966% | **---** | **---** | **---** | **---** | **---** | **---** | 0.946 | 1.453 |
| N2 | Genipin 1-gentiobioside | soluble | 2.244 | 22.762% | **---** | **---** | **---** | **---** | **---** | **---** | 0.471 | 1.190 |
| N3 | Ellagic acid | slightly soluble | 1.117 | 78.228% | **---** | **+++** | **---** | **---** | **---** | **---** | 0.863 | 2.346 |
| N4 | Isovitexin | slightly soluble | 0.414 | 90.306% | **---** | **---** | **---** | **---** | **---** | **---** | 0.481 | 1.970 |
| N5 | Quercetin | slightly soluble | 2.155 | 95.496% | **---** | **+++** | **---** | **+** | **-** | **-** | 0.929 | 8.284 |
| N6 | Obtusin | slightly soluble | 3.211 | 94.012% | **+++** | **-** | **---** | **+** | **---** | **-** | 0.360 | 8.465 |
| N7 | Kaempferol | slightly soluble | 2.656 | 97.861% | **---** | **+++** | **--** | **+** | **++** | **+** | 0.905 | 6.868 |
| N8 | Formononetin | almost insoluble | 3.233 | 97.256% | **---** | **+++** | **++** | **+** | **+++** | **++** | 0.547 | 5.497 |
| N9 | Aurantio-obtusin | slightly soluble | 3.078 | 97.842% | **++** | **++** | **---** | **+** | **---** | **-** | 0.412 | 7.987 |
| N10 | Isorhamnetin | slightly soluble | 2.541 | 96.235% | **---** | **+++** | **--** | **+** | **+** | **+** | 0.922 | 6.991 |
| N11 | Kaempferide | slightly soluble | 3.241 | 97.540% | **---** | **+++** | **+** | **++** | **++** | **+** | 0.850 | 5.176 |
| N12 | Galangin | slightly soluble | 3.260 | 98.593% | **---** | **+++** | **--** | **++** | **++** | **+** | 0.881 | 4.435 |
| N13 | Moupinamide | slightly soluble | 2.538 | 96.833% | **---** | **++** | **++** | **++** | **++** | **+** | 0.929 | 12.482 |
| N14 | Luteanin | slightly soluble | 2.421 | 79.695% | **--** | **--** | **---** | **---** | **---** | **--** | 0.366 | 11.800 |
| N15 | Scoulerine | slightly soluble | 2.034 | 88.666% | **+** | **-** | **--** | **---** | **++** | **---** | 0.856 | 18.087 |
| N16 | Tetrahydropalmatine | slightly soluble | 2.653 | 83.014% | **++** | **--** | **--** | **---** | **+** | **---** | 0.716 | 10.615 |
| N17 | Bicuculline | slightly soluble | 2.424 | 94.246% | **---** | **++** | **+++** | **+** | **+++** | **+++** | 0.073 | 14.084 |
| N18 | Chelidonine | slightly soluble | 2.614 | 89.732% | **---** | **+++** | **+++** | **--** | **+++** | **+++** | 0.221 | 17.024 |
| N19 | Coptisine | slightly soluble | 4.687 | 96.577% | **---** | **+++** | **--** | **---** | **+++** | **++** | 0.167 | 15.587 |
| N20 | Jatrorrhizine | soluble | 3.526 | 85.670% | **---** | **-** | **---** | **---** | **-** | **---** | 0.497 | 10.517 |
| N21 | Dihydrochelerythrine | insoluble | 4.971 | 92.225% | **--** | **+++** | **+++** | **++** | **+++** | **++** | 0.151 | 13.572 |
| N22 | Dehydrocorydaline | soluble | 4.399 | 79.770% | **--** | **---** | **---** | **---** | **--** | **---** | 0.470 | 8.761 |

Notes:

LogP: Octanol-water partition coefficient.optimal:0-3

Plasma protein binding.optimal:<90%.drugs with high protein-bound may have a low therapeutic index

Drug half-life:long half-life>3h;short half-life<3h

Blood components：high>15ml/min/kg;moderate:5-15ml/min/kg;low<5ml/min/kg;

For the classification endpoints , the prediction probability values are transformed into six symbols: 0-0.1(---), 0.1-0.3(--), 0.3-0.5(-), 0.5-0.7(+), 0.7-0.9(++), and 0.9-1.0(+++).
