# Supplementary material for: Elucidating the material basis and potential mechanisms of Ershiwuwei Lvxue Pill acting on rheumatoid arthritis by UPLC-Q-TOF/MS and network pharmacology
Source: PLoS One. 2022 Feb 7;17(2):e0262469. doi: 10.1371/journal.pone.0262469 (PMC8820630; doi:10.1371/journal.pone.0262469)
Supplement: S3 Table — (DOCX) [file pone.0262469.s006.docx]

S3 Table. Targets of potential bioactive compounds.

| **NO.** | **Molecule name** | **Target** | **Total** |
| --- | --- | --- | --- |
| N1 | chebulic acid | F10, IDO1, FOLH1, PTGES, JUN, MAPK14, BCL2, FUT4, STAT1, IL4, IL6 | 10 |
| N2 | Genipin 1-gentiobioside | CA2, CA1, SLC29A1, NEU4, BCL2L1, CYP19A1, MMP1, CASP3 | 8 |
| N3 | Ellagic acid | CYP19A1, EGFR, F2, CA2, PIM1, DRD4, ADORA1, MPO, PIK3R1, DAPK1, CA3, ALOX15, AKT1, NUAK1, HSPA5, NFKB1 | 16 |
| N4 | Isovitexin | TNF, ADORA1, IL2, EGFR, AKT1, CYP1B1, TP53, IL2RA | 7 |
| N5 | Quercetin | CYP19A1, EGFR, F2, CA2, PIM1, MPO, PIK3R1, KDR, ALK, AKT1, BACE1, CYP1B1, AXL, PIK3CG, AHR, CD40LG, IFNG, IL1A, IL1B, MAPK3, NFKB1, NR1L2, TGFB1, VEGFA | 24 |
| N6 | Obtusin | EGFR, ESR2, PIM1, ESR1, MCL1, BCL2, MMP1 | 7 |
| N7 | Kaempferol | NQO1, NOX4, CA2, AHR, CA12, ESRRA, CYP1B1, EGFR, IGF1R, F2, PIM1, MPO, PIK3R1, AKT1, ESR1, PTGS2, GSTA1, GSTM1, HSPA5 | 19 |
| N8 | Formononetin | IL2, CYP19A1, ALDH2, ESR1, CA7, EGFR, PPARA, PTGS1, TLR9, MAOB, CA2, OPRD1, IL4, IL2RA | 13 |
| N9 | Aurantio-obtusin | ESR2, EGFR, BCL2, PIM1, CYP19A1, MMP1, CYP1B1, STAT3, CASP3, AR, PIK3CG | 11 |
| N10 | Isorhamnetin | CA2, CYP1B1, NOX4, IGF1R, EGFR, CYP19A1, F2, PIM1, MPO, PIK3R1, AKT1, PIK3CG, AHR, ESR2, MPG, CD38 | 16 |
| N11 | Kaempferide | CYP1B1, CA2, MCL1, CYP19A1, NOX4, AHR, ESR2, EGFR, IGF1R, ALOX12, PIK3CG, PIM1, MPO, PIK3R1, AKT1, AR | 16 |
| N12 | Galangin | CYP1B1, CA2, ESR2, NOX4, PIM1, CSNK2A1, EGFR, IGF1R, CYP19A1, AHR, ESR1, PTGS2, IKBKB, PIK3CG | 14 |
| N13 | Moupinamide | MMP1, EGFR, PTGS2, TNF | 4 |
| N14 | Luteanin | NOS2, EGFR, TERT, PIM1, PTGS2, KDR, CDK2, GHSR, DPP9, EPHB4, JAK2, FAP, CHRM3, MAPK14, BRD4, JAK3, LIMK1, LIMK2 | 18 |
| N15 | Scoulerine | DRD1, DRD2, F3, DRD3, SIGMAR1, DRD5, DRD4, ESR1, EPHB4, NOX4, MMP1 | 11 |
| N16 | Tetrahydropalmatine | DRD1, CYP1A1, OPRD1, PTGS2, PTGER2, MAPK9, TGFBR1, PRKD2, SCD, JUN, ESR1, TUBB1, SIRT2, AHR, PIK3CA, EZR, JAK3 | 17 |
| N17 | Bicuculline | MAPK9, HTR3A, CASP3, MPO, IKBKB, PTGS2, CHRNA7, PIK3CA | 8 |
| N18 | Chelidonine | NOS2, CCL2, ROCK1, PDE5A, HTR1F, LCK, JAK2, JAK3, CTSK, HSP90B1, PIM2, CACNA1C, EGFR, MC4R, SSTR4, CASR, SSTR1, PRKDC | 18 |
| N19 | Coptisine | CA2, PIK3CG, BRAF, CYP11B1, TYMS, PIK3CA, CYP11B2, CHEK1, NQO2, MKNK1, EIF2AK2, CDK8, IKBKB, PTGS1, PTGS2, CYP17A1 | 16 |
| N20 | Jatrorrhizine | HTR3A, MMP1, DRD4, ROCK2, PIK3CG, MAP4K4, PDK1, CDC25B, IKBKB, TBXA2R, PIM1 | 11 |
| N21 | Dihydrochelerythrine | PIK3CA, CYP19A1, MAPK14, CASP3, CLK3, PIK3CG, PTGS2, AKT1, EGFR, DCK, MCL1, NOS2, BACE1, CHRM4, KCNH2, ESR2, CDK2 | 17 |
| N22 | Dehydrocorydaline | CYP1A1, PFKFB3, MAPK10, TBXAS1, IKBKB, ROCK2, OXTR, CDK2 | 8 |
