# Supplementary material for: Elucidating the material basis and potential mechanisms of Ershiwuwei Lvxue Pill acting on rheumatoid arthritis by UPLC-Q-TOF/MS and network pharmacology
Source: PLoS One. 2022 Feb 7;17(2):e0262469. doi: 10.1371/journal.pone.0262469 (PMC8820630; doi:10.1371/journal.pone.0262469)
Supplement: S4 Table — (DOCX) [file pone.0262469.s007.docx]

S4 Table. 361 RA-related targets were retrieved from GeneCards, CTD, and OMIM databases.

| **Gene Symbol** | **Description** | **Gene Symbol** | **Description** |
| --- | --- | --- | --- |
| IL6 | Interleukin 6 | GP1BB | Glycoprotein Ib Platelet Subunit Beta |
| HLA-DRB1 | Major Histocompatibility Complex, Class II, DR Beta 1 | CENPB | Centromere Protein B |
| TNF | Tumor Necrosis Factor | CCL21 | C-C Motif Chemokine Ligand 21 |
| PTPN22 | Protein Tyrosine Phosphatase Non-Receptor Type 22 | MS4A1 | Membrane Spanning 4-Domains A1 |
| IL2RA | Interleukin 2 Receptor Subunit Alpha | GZMB | Granzyme B |
| IL10 | Interleukin 10 | HLA-DOA | Major Histocompatibility Complex, Class II, DO Alpha |
| STAT4 | Signal Transducer And Activator Of Transcription 4 | PTHLH | Parathyroid Hormone Like Hormone |
| PADI4 | Peptidyl Arginine Deiminase 4 | TAC1 | Tachykinin Precursor 1 |
| MIF | Macrophage Migration Inhibitory Factor | CCR7 | C-C Motif Chemokine Receptor 7 |
| IL1B | Interleukin 1 Beta | NOS2 | Nitric Oxide Synthase 2 |
| IRF5 | Interferon Regulatory Factor 5 | HTR2A | 5-Hydroxytryptamine Receptor 2A |
| HLA-DQA1 | Major Histocompatibility Complex, Class II, DQ Alpha 1 | CTSB | Cathepsin B |
| HLA-B | Major Histocompatibility Complex, Class I, B | CASP8 | Caspase 8 |
| LTA | Lymphotoxin Alpha | PIK3CG | Phosphatidylinositol-4,5-Bisphosphate 3-Kinase Catalytic Subunit Gamma |
| TLR4 | Toll Like Receptor 4 | HRAS | HRas Proto-Oncogene, GTPase |
| TNFRSF1A | TNF Receptor Superfamily Member 1A | DKK1 | Dickkopf WNT Signaling Pathway Inhibitor 1 |
| CRP | C-Reactive Protein | PPP1CB | Protein Phosphatase 1 Catalytic Subunit Beta |
| CTLA4 | Cytotoxic T-Lymphocyte Associated Protein 4 | GATA3 | GATA Binding Protein 3 |
| LACC1 | Laccase Domain Containing 1 | CXCR2 | C-X-C Motif Chemokine Receptor 2 |
| IL17A | Interleukin 17A | PTGS1 | Prostaglandin-Endoperoxide Synthase 1 |
| MMP3 | Matrix Metallopeptidase 3 | TP53 | Tumor Protein P53 |
| HLA-DQB1 | Major Histocompatibility Complex, Class II, DQ Beta 1 | LEP | Leptin |
| ACP5 | Acid Phosphatase 5, Tartrate Resistant | IL21R | Interleukin 21 Receptor |
| CIITA | Class II Major Histocompatibility Complex Transactivator | AKT1 | AKT Serine/Threonine Kinase 1 |
| MIR146A | MicroRNA 146a | CXCL2 | C-X-C Motif Chemokine Ligand 2 |
| NLRP1 | NLR Family Pyrin Domain Containing 1 | STAT6 | Signal Transducer And Activator Of Transcription 6 |
| SAA1 | Serum Amyloid A1 | MTOR | Mechanistic Target Of Rapamycin Kinase |
| IL18 | Interleukin 18 | CD2 | CD2 Molecule |
| IL1RN | Interleukin 1 Receptor Antagonist | EGFR | Epidermal Growth Factor Receptor |
| NOD2 | Nucleotide Binding Oligomerization Domain Containing 2 | CCL13 | C-C Motif Chemokine Ligand 13 |
| CCR6 | C-C Motif Chemokine Receptor 6 | JAK2 | Janus Kinase 2 |
| TNIP1 | TNFAIP3 Interacting Protein 1 | COL5A1 | Collagen Type V Alpha 1 Chain |
| TNFRSF1B | TNF Receptor Superfamily Member 1B | JAK1 | Janus Kinase 1 |
| TNFSF11 | TNF Superfamily Member 11 | CXCL13 | C-X-C Motif Chemokine Ligand 13 |
| IFNG | Interferon Gamma | CCL7 | C-C Motif Chemokine Ligand 7 |
| CXCL8 | C-X-C Motif Chemokine Ligand 8 | BAX | BCL2 Associated X, Apoptosis Regulator |
| SLC22A4 | Solute Carrier Family 22 Member 4 | JAK3 | Janus Kinase 3 |
| MMP1 | Matrix Metallopeptidase 1 | PIK3CD | Phosphatidylinositol-4,5-Bisphosphate 3-Kinase Catalytic Subunit Delta |
| TTR | Transthyretin | BCL2L11 | BCL2 Like 11 |
| IL1A | Interleukin 1 Alpha | CXCL3 | C-X-C Motif Chemokine Ligand 3 |
| PSTPIP1 | Proline-Serine-Threonine Phosphatase Interacting Protein 1 | MTR | 5-Methyltetrahydrofolate-Homocysteine Methyltransferase |
| IL2RB | Interleukin 2 Receptor Subunit Beta | TGFA | Transforming Growth Factor Alpha |
| CCL2 | C-C Motif Chemokine Ligand 2 | CDK4 | Cyclin Dependent Kinase 4 |
| IL1R1 | Interleukin 1 Receptor Type 1 | AKT2 | AKT Serine/Threonine Kinase 2 |
| FLG | Filaggrin | IL1RAP | Interleukin 1 Receptor Accessory Protein |
| CD244 | CD244 Molecule | TLR6 | Toll Like Receptor 6 |
| NFKBIL1 | NFKB Inhibitor Like 1 | MCL1 | MCL1 Apoptosis Regulator, BCL2 Family Member |
| MIR155 | MicroRNA 155 | SOCS1 | Suppressor Of Cytokine Signaling 1 |
| MEFV | MEFV Innate Immuity Regulator, Pyrin | MAPK3 | Mitogen-Activated Protein Kinase 3 |
| TNFRSF11B | TNF Receptor Superfamily Member 11b | SELL | L-selectin |
| MMP13 | Matrix Metallopeptidase 13 | RELA | Transcription factor p65 |
| PTGS2 | Prostaglandin-Endoperoxide Synthase 2 | BAK1 | Bcl-2 homologous antagonist/killer |
| TGFB1 | Transforming Growth Factor Beta 1 | CASP9 | Caspase-9 |
| HLA-DPB1 | Major Histocompatibility Complex, Class II, DP Beta 1 | CXCR4 | C-X-C chemokine receptor type 4 |
| IL4 | Interleukin 4 | SOD2 | Superoxide dismutase [Mn], mitochondrial |
| S100A12 | S100 Calcium Binding Protein A12 | ALB | Afamin |
| TLR2 | Toll Like Receptor 2 | DDIT3 | DNA damage-inducible transcript 3 protein |
| COMP | Cartilage Oligomeric Matrix Protein | GSK3B | Glycogen synthase kinase-3 beta |
| IL2 | Interleukin 2 | DUSP1 | Dual specificity protein phosphatase 1 |
| FAS | Fas Cell Surface Death Receptor | NFE2L2 | Nuclear factor erythroid 2-related factor 2 |
| VEGFA | Vascular Endothelial Growth Factor A | PCNA | PCNA-associated factor |
| ICAM1 | Intercellular Adhesion Molecule 1 | SOD1 | Superoxide dismutase [Cu-Zn] |
| CSF1 | Colony Stimulating Factor 1 | ISG15 | Ubiquitin-like protein ISG15 |
| IL15 | Interleukin 15 | SLC7A11 | Cystine/glutamate transporter |
| COL2A1 | Collagen Type II Alpha 1 Chain | TXNRD1 | Thioredoxin reductase 1, cytoplasmic |
| SLC11A1 | Solute Carrier Family 11 Member 1 | CCND1 | G1/S-specific cyclin-D1 |
| MIR132 | MicroRNA 132 | TNFSF10 | Tumor necrosis factor ligand superfamily member 10 |
| CD40LG | CD40 Ligand | NFKBIA | NF-kappa-B inhibitor alpha |
| MMP9 | Matrix Metallopeptidase 9 | TXNIP | Thioredoxin-interacting protein |
| CSF2 | Colony Stimulating Factor 2 | CASP3 | Caspase-3 |
| FCRL3 | Fc Receptor Like 3 | BIRC3 | Baculoviral IAP repeat-containing protein 3 |
| CCL5 | C-C Motif Chemokine Ligand 5 | OAS1 | Baculoviral IAP repeat-containing protein 3 |
| TNFRSF11A | TNF Receptor Superfamily Member 11a | BCL2L1 | Bcl-2-like protein 1 |
| SELE | Selectin E | HPGD | 15-hydroxyprostaglandin dehydrogenase [NAD(+)] |
| MIR150 | MicroRNA 150 | HSPA1A | Heat shock 70 kDa protein 1A |
| VCAM1 | Vascular Cell Adhesion Molecule 1 | MYC | Myc proto-oncogene protein |
| IL23R | Interleukin 23 Receptor | BCL2A1 | Bcl-2-related protein A1 |
| TNFSF13B | TNF Superfamily Member 13b | MKI67 | MKI67 FHA domain-interacting nucleolar phosphoprotein |
| HLA-C | Major Histocompatibility Complex, Class I, C | MMP2 | 72 kDa type IV collagenase |
| CD79A | CD79a Molecule | ISG20 | Interferon-stimulated gene 20 kDa protein |
| TIMP1 | TIMP Metallopeptidase Inhibitor 1 | COL1A1 | Collagen alpha-1(I) chain |
| CCL3 | C-C Motif Chemokine Ligand 3 | IL12A | Interleukin-12 subunit alpha |
| PTPN2 | Protein Tyrosine Phosphatase Non-Receptor Type 2 | IL5 | Interleukin-5 receptor subunit alpha |
| NLRP3 | NLR Family Pyrin Domain Containing 3 | APP | Amyloid-beta precursor protein |
| CD247 | CD247 Molecule | OGG1 | N-glycosylase/DNA lyase |
| FASLG | Fas Ligand | VIM | Vimentin |
| IL23A | Interleukin 23 Subunit Alpha | CXCL10 | C-X-C motif chemokine 10 |
| TRAF1 | TNF Receptor Associated Factor 1 | EGR1 | Early growth response protein 1 |
| FCGR3A | Fc Fragment Of IgG Receptor IIIa | NR3C1 | Glucocorticoid receptor |
| CD28 | CD28 Molecule | ATF6 | Cyclic AMP-dependent transcription factor ATF-6 alpha |
| CXCL12 | C-X-C Motif Chemokine Ligand 12 | ACKR3 | Atypical chemokine receptor 3 |
| IL17RA | Interleukin 17 Receptor A | ABCB1 | ATP-dependent translocase ABCB1 |
| S100A9 | S100 Calcium Binding Protein A9 | CDKN1A | Cyclin-dependent kinase inhibitor 1 |
| IL6R | Interleukin 6 Receptor | PLA2G4A | Cytosolic phospholipase A2 |
| JUN | Jun Proto-Oncogene, AP-1 Transcription Factor Subunit | IL12B | Interleukin-12 subunit beta |
| FOXP3 | Forkhead Box P3 | MAPK1 | Mitogen-activated protein kinase 1 |
| ANKH | ANKH Inorganic Pyrophosphate Transport Regulator | CHUK | Inhibitor of nuclear factor kappa-B kinase subunit alpha |
| HLA-DMA | Major Histocompatibility Complex, Class II, DM Alpha | GPX1 | Glutathione peroxidase 1 |
| ADAM17 | ADAM Metallopeptidase Domain 17 | IGFBP3 | Insulin-like growth factor-binding protein 3 |
| TNFAIP6 | TNF Alpha Induced Protein 6 | CD44 | CD44 antigen |
| MMP12 | Matrix Metallopeptidase 12 | TRP53 | Thiamine-triphosphatase |
| BGLAP | Bone Gamma-Carboxyglutamate Protein | FKBP5 | Peptidyl-prolyl cis-trans isomerase FKBP5 |
| C4A | Complement C4A (Rodgers Blood Group) | BID | BH3-interacting domain death agonist |
| ITGB2 | Integrin Subunit Beta 2 | BAD | Bcl2-associated agonist of cell death |
| ANKRD55 | Ankyrin Repeat Domain 55 | BCL3 | B-cell lymphoma 3 protein |
| HLA-DMB | Major Histocompatibility Complex, Class II, DM Beta | GCLC | Glutamate--cysteine ligase catalytic subunit |
| CAT | Catalase | MARCKS | MARCKS-related protein |
| HMGB1 | High Mobility Group Box 1 | TNFRSF10B | Tumor necrosis factor receptor superfamily member 10B |
| CCL20 | C-C Motif Chemokine Ligand 20 | FOXO1 | Forkhead box protein O1 |
| CXCL1 | C-X-C Motif Chemokine Ligand 1 | CCNA2 | Cyclin-A2 |
| IL11 | Interleukin 11 | CASP7 | Caspase-7 |
| CD80 | CD80 Molecule | XBP1 | X-box-binding protein 1 |
| CTSK | Cathepsin K | HSD11B2 | Corticosteroid 11-beta-dehydrogenase isozyme 2 |
| FOS | Fos Proto-Oncogene, AP-1 Transcription Factor Subunit | HMOX1 | Heme oxygenase 1 |
| MICA | MHC Class I Polypeptide-Related Sequence A | BIRC5 | Baculoviral IAP repeat-containing protein 5 |
| HLA-DRA | Major Histocompatibility Complex, Class II, DR Alpha | CDH1 | Fizzy-related protein homolog |
| HLA-DQA2 | Major Histocompatibility Complex, Class II, DQ Alpha 2 | BBC3 | Bcl-2-binding component 3, isoforms 1/2 |
| ERAP1 | Endoplasmic Reticulum Aminopeptidase 1 | CEBPB | Bcl-2-binding component 3, isoforms 1/2 |
| SERPINH1 | Serpin Family H Member 1 | CTNNB1 | Catenin beta-1 |
| TNFSF13 | TNF Superfamily Member 13 | CYCS | Cytochrome c |
| REL | REL Proto-Oncogene, NF-KB Subunit | ODC1 | Ornithine decarboxylase |
| CXCL5 | C-X-C Motif Chemokine Ligand 5 | CCL4 | C-C motif chemokine 4-like |
| TNFAIP3 | TNF Alpha Induced Protein 3 | IFI6 | Interferon alpha-inducible protein 6 |
| TEK | TEK Receptor Tyrosine Kinase | SLC22A7 | Solute carrier family 22 member 7 |
| CD86 | CD86 Molecule | TJP1 | Tight junction protein ZO-1 |
| ANGPT1 | Angiopoietin 1 | PMAIP1 | Phorbol-12-myristate-13-acetate-induced protein 1 |
| PTH | Parathyroid Hormone | SAT1 | Diamine acetyltransferase 1 |
| FSTL1 | Follistatin Like 1 | GBP1 | Guanylate-binding protein 1 |
| ITGAL | Integrin Subunit Alpha L | PER2 | Period circadian protein homolog 2 |
| TAGAP | T Cell Activation RhoGTPase Activating Protein | TIMP3 | Metalloproteinase inhibitor 3 |
| CHI3L1 | Chitinase 3 Like 1 | EEF1A1 | Elongation factor 1-alpha 1 |
| TFRC | Transferrin Receptor | TIMP2 | Metalloproteinase inhibitor 2 |
| HLA-DRB5 | Major Histocompatibility Complex, Class II, DR Beta 5 | HIF1A | Hypoxia-inducible factor 1-alpha |
| IL6ST | Interleukin 6 Signal Transducer | C3 | Complement C3 |
| FLT1 | Fms Related Tyrosine Kinase 1 | CDK1 | Cyclin-dependent kinase 1 |
| HSPA5 | Heat Shock Protein Family A (Hsp70) Member 5 | NQO1 | NAD(P)H dehydrogenase [quinone] 1 |
| CTSL | Cathepsin L | BST2 | Bone marrow stromal antigen 2 |
| IL37 | Interleukin 37 | IKBKB | Inhibitor of nuclear factor kappa-B kinase subunit beta |
| MICB | MHC Class I Polypeptide-Related Sequence B | PCK1 | Phosphoenolpyruvate carboxykinase, cytosolic [GTP] |
| CARD8 | Caspase Recruitment Domain Family Member 8 | ABCC4 | Multidrug resistance-associated protein 4 |
| STAT3 | Signal Transducer And Activator Of Transcription 3 | HSPD1 | 60 kDa heat shock protein, mitochondrial |
| C5orf30 | Chromosome 5 Open Reading Frame 30 | HSPB1 | Heat shock protein beta-1 |
| PRTN3 | Proteinase 3 | ATF3 | Cyclic AMP-dependent transcription factor ATF-3 |
| TNFSF4 | TNF Superfamily Member 4 | CLGN | Calmegin |
| CCR2 | C-C Motif Chemokine Receptor 2 | TNFAIP2 | Tumor necrosis factor alpha-induced protein 2 |
| IL13 | Interleukin 13 | NR2F2 | COUP transcription factor 2 |
| CCN2 | Cellular Communication Network Factor 2 | ERN1 | Serine/threonine-protein kinase/endoribonuclease IRE1 |
| NR4A2 | Nuclear Receptor Subfamily 4 Group A Member 2 | CLU | Clusterin |
| FOLR2 | Folate Receptor Beta | CCNB1 | E3 ubiquitin-protein ligase CCNB1IP1 |
| OLAH | Oleoyl-ACP Hydrolase | ATF4 | Cyclic AMP-dependent transcription factor ATF-4 |
| CSF1R | Colony Stimulating Factor 1 Receptor | PLPP3 | Phospholipid phosphatase 3 |
| CXCL6 | C-X-C Motif Chemokine Ligand 6 | EGF | Stabilin-2 |
| DEK | DEK Proto-Oncogene | TGFB2 | Transforming growth factor beta-2 proprotein |
| ANXA1 | Annexin A1 | NGFR | Tumor necrosis factor receptor superfamily member 16 |
| LTF | Lactotransferrin | EFEMP1 | EGF-containing fibulin-like extracellular matrix protein 1 |
| SPP1 | Secreted Phosphoprotein 1 | AIFM1 | Apoptosis-inducing factor 1, mitochondrial |
| HAPLN1 | Hyaluronan And Proteoglycan Link Protein 1 | SLC20A1 | Sodium-dependent phosphate transporter 1 |
| SUPT20H | SPT20 Homolog, SAGA Complex Component | GSTP1 | Glutathione S-transferase P |
| STAT1 | Signal Transducer And Activator Of Transcription 1 | IER3 | Radiation-inducible immediate-early gene IEX-1 |
| H19 | H19 Imprinted Maternally Expressed Transcript | PIM1 | Serine/threonine-protein kinase pim-1 |
| NFKB1 | Nuclear Factor Kappa B Subunit 1 | SLC22A6 | Solute carrier family 22 member 6 |
| UCA1 | Urothelial Cancer Associated 1 | SLC3A2 | 4F2 cell-surface antigen heavy chain |
| MPO | Myeloperoxidase | TICAM1 | TIR domain-containing adapter molecule 1 |
| COMT | Catechol-O-Methyltransferase | CD55 | Complement decay-accelerating factor |
| PRKCD | Protein Kinase C Delta | PLAT | Tissue-type plasminogen activator |
| VDR | Vitamin D Receptor | BECN1 | Beclin-1 |
| PTGES | Prostaglandin E Synthase | GSTM1 | Glutathione S-transferase Mu 1 |
| CAV1 | Caveolin 1 | CASP1 | Caspase-1 |
| BCL2 | BCL2 Apoptosis Regulator | CYP1A1 | Cytochrome P450 1A1 |
| PTPRC | Protein Tyrosine Phosphatase Receptor Type C | PPARG | Peroxisome proliferator-activated receptor gamma |
| RELB | RELB Proto-Oncogene, NF-KB Subunit | MET | Hepatocyte growth factor receptor |
| DCLRE1C | DNA Cross-Link Repair 1C | XIAP | E3 ubiquitin-protein ligase XIAP |
| TLR9 | Toll Like Receptor 9 | KDR | Vascular endothelial growth factor receptor 2 |
| MYD88 | MYD88 Innate Immune Signal Transduction | CDK2 | Cyclin-dependent kinase 2 |
| MAPK14 | Mitogen-Activated Protein Kinase 14 | SELP | Selenoprotein P |
| VIP | Vasoactive Intestinal Peptide | F3 | Cytochrome P450 4F3 |
| CCL17 | C-C Motif Chemokine Ligand 17 | GPX2 | Glutathione peroxidase 2 |
| BTNL2 | Butyrophilin Like 2 | IGF1R | Insulin-like growth factor 1 receptor |
| MRAP | Melanocortin 2 Receptor Accessory Protein | ITM2A | Integral membrane protein 2A |
| NPY | Neuropeptide Y | GSTA1 | Glutathione S-transferase A1 |
| TBX1 | T-Box Transcription Factor 1 |  |  |
