# Supplementary material for: Elucidating the material basis and potential mechanisms of Ershiwuwei Lvxue Pill acting on rheumatoid arthritis by UPLC-Q-TOF/MS and network pharmacology
Source: PLoS One. 2022 Feb 7;17(2):e0262469. doi: 10.1371/journal.pone.0262469 (PMC8820630; doi:10.1371/journal.pone.0262469)
Supplement: S5 Table — (DOCX) [file pone.0262469.s008.docx]

S5 Table. Target information of ELP in the treatment of RA.

| **No.** | **Uniprot ID** | **Common name** | **Target** | **Betweenness Centrality** | **Closeness Centrality** | **Degree** |
| --- | --- | --- | --- | --- | --- | --- |
| 1 | P05231 | IL6 | Interleukin-6 | 0.049120 | 0.918367 | 41 |
| 2 | P01375 | TNF | TNF-alpha | 0.025298 | 0.882353 | 39 |
| 3 | P04637 | TP53 | Tumour suppressor p53/oncoprotein Mdm2 | 0.052201 | 0.865385 | 38 |
| 4 | P31749 | AKT1 | Serine/threonine-protein kinase AKT | 0.025356 | 0.865385 | 38 |
| 5 | P05412 | JUN | Proto-oncogene c-JUN | 0.023715 | 0.849057 | 37 |
| 6 | P15498 | VEGFA | Vascular Endothelial Growth Factor A | 0.020028 | 0.849057 | 37 |
| 7 | P27361 | MAPK3 | Mitogen-Activated Protein Kinase 3 | 0.020789 | 0.849057 | 37 |
| 8 | P40763 | STAT3 | Signal transducer and activator of transcription 3 | 0.023969 | 0.849057 | 37 |
| 9 | P01584 | IL1B | Interleukin 1 Beta | 0.049214 | 0.833333 | 36 |
| 10 | P35354 | PTGS2 | Cyclooxygenase-2 | 0.021122 | 0.818182 | 35 |
| 11 | Q16539 | MAPK14 | MAP kinase p38 alpha | 0.016474 | 0.818182 | 35 |
| 12 | P42574 | CASP3 | Caspase-3 | 0.012748 | 0.803571 | 34 |
| 13 | P00533 | EGFR | Epidermal growth factor receptor erbB1 | 0.019432 | 0.775862 | 32 |
| 14 | P13500 | CCL2 | C-C motif chemokine 2 | 0.009522 | 0.775862 | 32 |
| 15 | Q07817 | BCL2L1 | Apoptosis regulator Bcl-X | 0.012286 | 0.775862 | 32 |
| 16 | P05112 | IL4 | Interleukin-4 | 0.005210 | 0.762712 | 31 |
| 17 | P60568 | IL2 | Interleukin-2 | 0.005302 | 0.762712 | 31 |
| 18 | P01579 | IFNG | Interferon Gamma | 0.004670 | 0.750000 | 30 |
| 19 | P19838 | NFKB1 | Nuclear Factor Kappa B Subunit 1 | 0.008391 | 0.750000 | 30 |
| 20 | P42224 | STAT1 | Signal transducer and activator of transcription 1-alpha/beta | 0.006744 | 0.750000 | 30 |
| 21 | O60674 | JAK2 | Tyrosine-protein kinase JAK2 | 0.011075 | 0.725806 | 28 |
| 22 | P29965 | CD40LG | CD40 Ligand | 0.004932 | 0.703125 | 26 |
| 23 | Q9NR96 | TLR9 | Toll-like receptor (TLR7/TLR9) | 0.001946 | 0.703125 | 26 |
| 24 | Q07820 | MCL1 | Induced myeloid leukemia cell differentiation protein Mcl-1 | 0.008793 | 0.681818 | 25 |
| 25 | P01137 | TGFB1 | Transforming Growth Factor Beta 1 | 0.001667 | 0.681818 | 24 |
| 26 | P05164 | MPO | Myeloperoxidase | 0.013784 | 0.681818 | 24 |
| 27 | P35968 | KDR | Vascular endothelial growth factor receptor 2 | 0.003548 | 0.681818 | 24 |
| 28 | P35228 | NOS2 | Nitric oxide synthase, inducible | 0.001344 | 0.671642 | 23 |
| 29 | P03956 | MMP1 | Matrix metalloproteinase 1 | 0.001868 | 0.652174 | 21 |
| 30 | P52333 | JAK3 | Tyrosine-protein kinase JAK3 | 0.002495 | 0.642857 | 21 |
| 31 | P01583 | IL1A | Interleukin 1 Alpha | 0.002160 | 0.633803 | 19 |
| 32 | P08069 | IGF1R | Insulin-like growth factor I receptor | 0.001930 | 0.633803 | 19 |
| 33 | P01589 | IL2RA | Interleukin-2 receptor subunit alpha | 0.000768 | 0.616438 | 18 |
| 34 | O14920 | IKBKB | Inhibitor of nuclear factor kappa B kinase beta subunit | 0.001156 | 0.616438 | 17 |
| 35 | P11021 | HSPA5 | Heat Shock Protein Family A (Hsp70) Member 5 | 0.000053 | 0.592105 | 15 |
| 36 | P13726 | F3 | Coagulation factor VII/tissue factor | 0.000251 | 0.600000 | 15 |
| 37 | P15559 | NQO1 | Quinone reductase 1 | 0.010894 | 0.592105 | 14 |
| 38 | P24941 | CDK2 | Cyclin-dependent kinase 2 | 0.000472 | 0.576923 | 13 |
| 39 | P10415 | BCL2 | Apoptosis regulator Bcl-2 | 0.001396 | 0.562500 | 12 |
| 40 | P43235 | CTSK | Cathepsin K | 0.001665 | 0.569620 | 12 |
| 41 | P48736 | PIK3CG | PI3-kinase p110-gamma subunit | 0.000436 | 0.555556 | 10 |
| 42 | O14684 | PTGES | Prostaglandin E synthase | 0.000000 | 0.529412 | 7 |
| 43 | P04798 | CYP1A1 | Cytochrome P450 1A1 | 0.003097 | 0.542169 | 7 |
| 44 | P09488 | GSTM1 | Glutathione S-transferase Mu 1 | 0.001034 | 0.505618 | 5 |
| 45 | P11309 | PIM1 | Serine/threonine-protein kinase PIM1 | 0.000000 | 0.517241 | 5 |
| 46 | P08263 | GSTA1 | Glutathione S-transferase A1 | 0.000533 | 0.483871 | 4 |
