# Supplementary material for: Elucidating the material basis and potential mechanisms of Ershiwuwei Lvxue Pill acting on rheumatoid arthritis by UPLC-Q-TOF/MS and network pharmacology
Source: PLoS One. 2022 Feb 7;17(2):e0262469. doi: 10.1371/journal.pone.0262469 (PMC8820630; doi:10.1371/journal.pone.0262469)
Supplement: S6 Table — (DOCX) [file pone.0262469.s009.docx]

S6 Table. Results of molecular docking simulation.

| **Molecule name** | **IL6** | **TNF** | **TP53** | **AKT1** | **JUN** | **VEGFA** | **MAPK3** | **STAT3** | **IL1B** | **PTGS2** |
| --- | --- | --- | --- | --- | --- | --- | --- | --- | --- | --- |
| **PDB ID** | **(1ALU)** | **(2AZ5)** | **(6GGC)** | **(1UNQ)** | **(1T2K)** | **(4QAF)** | **(4QTB)** | **(6NJS)** | **(1L2H)** | **(5KIR)** |
| Chebulic acid | 6.636 | 6.146 | 6.944 | 6.944 | 3.761 | 6.593 | 6.886 | 7.368 | 6.446 | 8.507 |
| Genipin 1-gentiobioside | 4.620 | 6.198 | 5.824 | 5.824 | 4.396 | 5.748 | 7.888 | 6.648 | 5.360 | 7.197 |
| Ellagic acid | 5.906 | 6.053 | 5.915 | 5.915 | 5.778 | 7.974 | 8.143 | 9.065 | 6.957 | 7.910 |
| Isovitexin | 3.171 | 6.674 | 6.638 | 6.638 | 4.962 | 7.843 | 8.502 | 8.846 | 6.289 | 7.878 |
| Quercetin | 5.848 | 6.976 | 6.342 | 6.342 | 4.965 | 7.341 | 9.484 | 8.342 | 6.060 | 7.924 |
| Obtusin | 5.109 | 5.991 | 4.548 | 4.548 | 4.937 | 7.436 | 7.450 | 6.683 | 4.270 | 7.953 |
| Kaempferol | 5.258 | 6.388 | 5.703 | 5.703 | 5.196 | 8.087 | 8.680 | 7.432 | 5.733 | 7.824 |
| Formononetin | 5.805 | 6.327 | 6.248 | 6.248 | 4.587 | 7.386 | 5.332 | 7.434 | 6.162 | 7.113 |
| Aurantio-obtusin | 4.882 | 6.339 | 6.805 | 6.805 | 5.023 | 7.438 | 7.511 | 7.027 | 5.506 | 8.146 |
| Isorhamnetin | 5.471 | 6.679 | 6.084 | 6.084 | 5.235 | 7.346 | 8.440 | 7.936 | 5.925 | 8.200 |
| Kaempferide | 5.346 | 6.432 | 6.025 | 6.025 | 5.265 | 7.693 | 7.368 | 7.139 | 5.912 | 8.186 |
| Galangin | 5.421 | 6.885 | 5.721 | 5.721 | 5.318 | 7.366 | 8.554 | 7.864 | 5.998 | 8.604 |
| Moupinamide | 4.679 | 5.092 | 5.515 | 5.515 | 5.167 | 6.803 | 6.211 | 6.540 | 4.493 | 6.889 |
| Luteanin | 3.505 | 6.096 | 4.256 | 4.256 | 3.576 | 4.427 | 6.318 | 5.611 | 4.923 | 7.483 |
| Scoulerine | 5.050 | 5.612 | 5.838 | 5.838 | 4.027 | 7.075 | 5.789 | 5.385 | 5.315 | 7.018 |
| Tetrahydropalmatine | 3.353 | 5.587 | 4.484 | 4.484 | 3.427 | 5.434 | 5.014 | 5.562 | 4.532 | 7.245 |
| Bicuculline | 3.522 | 5.902 | 4.963 | 4.963 | 4.654 | 6.416 | 6.837 | 7.083 | 5.299 | 5.987 |
| Chelidonine | 3.925 | 6.811 | 5.190 | 5.190 | 4.744 | 5.595 | 7.419 | 5.754 | 6.253 | 6.029 |
| Coptisine | 4.040 | 6.589 | 4.033 | 4.033 | 2.979 | 7.403 | 7.756 | 5.414 | 4.390 | 8.316 |
| Jatrorrhizine | 4.540 | 6.167 | 5.133 | 5.133 | 2.453 | 6.923 | 6.077 | 6.057 | 3.948 | 8.225 |
| Dihydrochelerythrine | 0.000 | 6.374 | 4.402 | 4.402 | 4.381 | 5.272 | 5.230 | 5.101 | 0.000 | 7.616 |
| Dehydrocorydaline | 3.645 | 6.605 | 4.222 | 4.222 | 3.326 | 6.659 | 5.182 | 5.338 | 4.697 | 7.880 |

Docking score represents a negative logarithm of experimental dissociation/inhibition constant value (pKd/pKi).
