# Supplementary material for: Elucidating the material basis and potential mechanisms of Ershiwuwei Lvxue Pill acting on rheumatoid arthritis by UPLC-Q-TOF/MS and network pharmacology
Source: PLoS One. 2022 Feb 7;17(2):e0262469. doi: 10.1371/journal.pone.0262469 (PMC8820630; doi:10.1371/journal.pone.0262469)
Supplement: S7 Table — (DOCX) [file pone.0262469.s010.docx]

S7 Table. Target information and native docking validation of TNF, VEGFA, MAPK3, STAT3 and PTGS2.

| Title | PDB ID | Self-ligand | Docking score | Affinity |
| --- | --- | --- | --- | --- |
| TNF | 2AZ5 | 307 | 8.199 | Kd: min: 3, max: 1.30e+4 (nM) from 12 assay(s) |
| VEGFA | 4QAF | OMA | 5.012 |  |
| MAPK3 | 4QTB | 38Z | 8.395 | Kd: 213 (nM) from 1 assay(s) |
| STAT3 | 6NJS | KQV | 10.941 |  |
| PTGS2 | 5KIR | RCX | 9.036 | IC50: min: 1, max: 3400 (nM) from 44 assay(s) |
