# Supplementary figures and images for: Elucidating the material basis and potential mechanisms of Ershiwuwei Lvxue Pill acting on rheumatoid arthritis by UPLC-Q-TOF/MS and network pharmacology
Source: PLoS One. 2022 Feb 7;17(2):e0262469. doi: 10.1371/journal.pone.0262469 (PMC8820630; doi:10.1371/journal.pone.0262469)

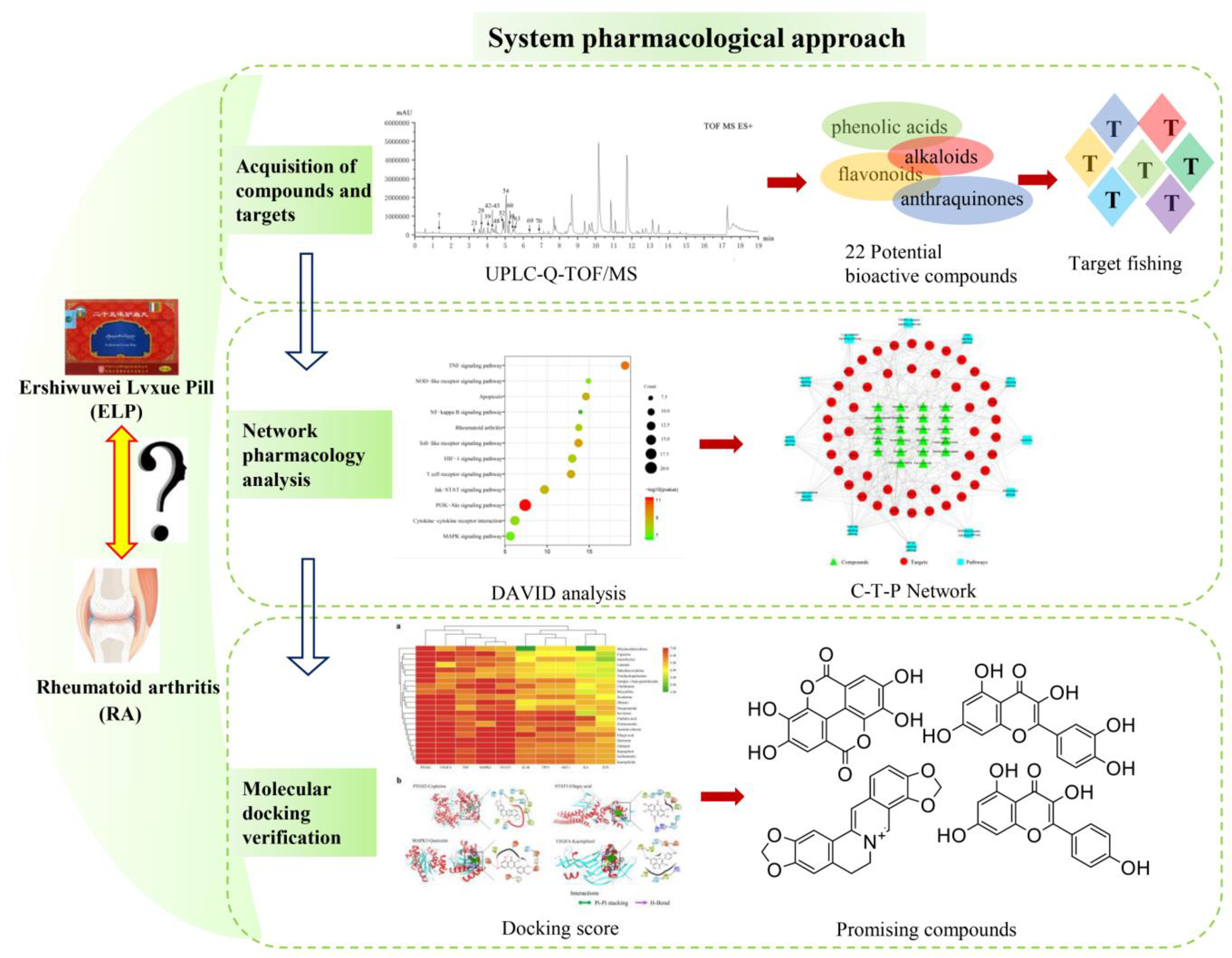

Supplement: S1 Graphical abstract — (TIF) [file pone.0262469.s011.tif]
